# Supplementary material for: Complex haploinsufficiency in pluripotent cells yields somatic cells with DNA methylation abnormalities and pluripotency induction defects
Source: Stem Cell Reports. 2023 Oct 12;18(11):2174–89. doi: 10.1016/j.stemcr.2023.09.009 (PMC10679652; doi:10.1016/j.stemcr.2023.09.009)
Supplement: Document S1. Figures S1–S6 and Table S2 [file mmc1.pdf]

Stem Cell Reports, Volume *18*

Supplemental Information

Complex haploinsufficiency in pluripotent cells yields somatic cells with DNA methylation abnormalities and pluripotency induction defects

Rachel Lasry, Noam Maoz, Albert W. Cheng, Nataly Yom Tov, Elisabeth Kulenkampff, Meir Azagury, Hui Yang, Cora Ople, Styliani Markoulaki, Dina A. Faddah, Kirill Makedonski, Dana Orzech, Ofra Sabag, Rudolf Jaenisch, and Yosef Buganim

RESULTS

Figure S1

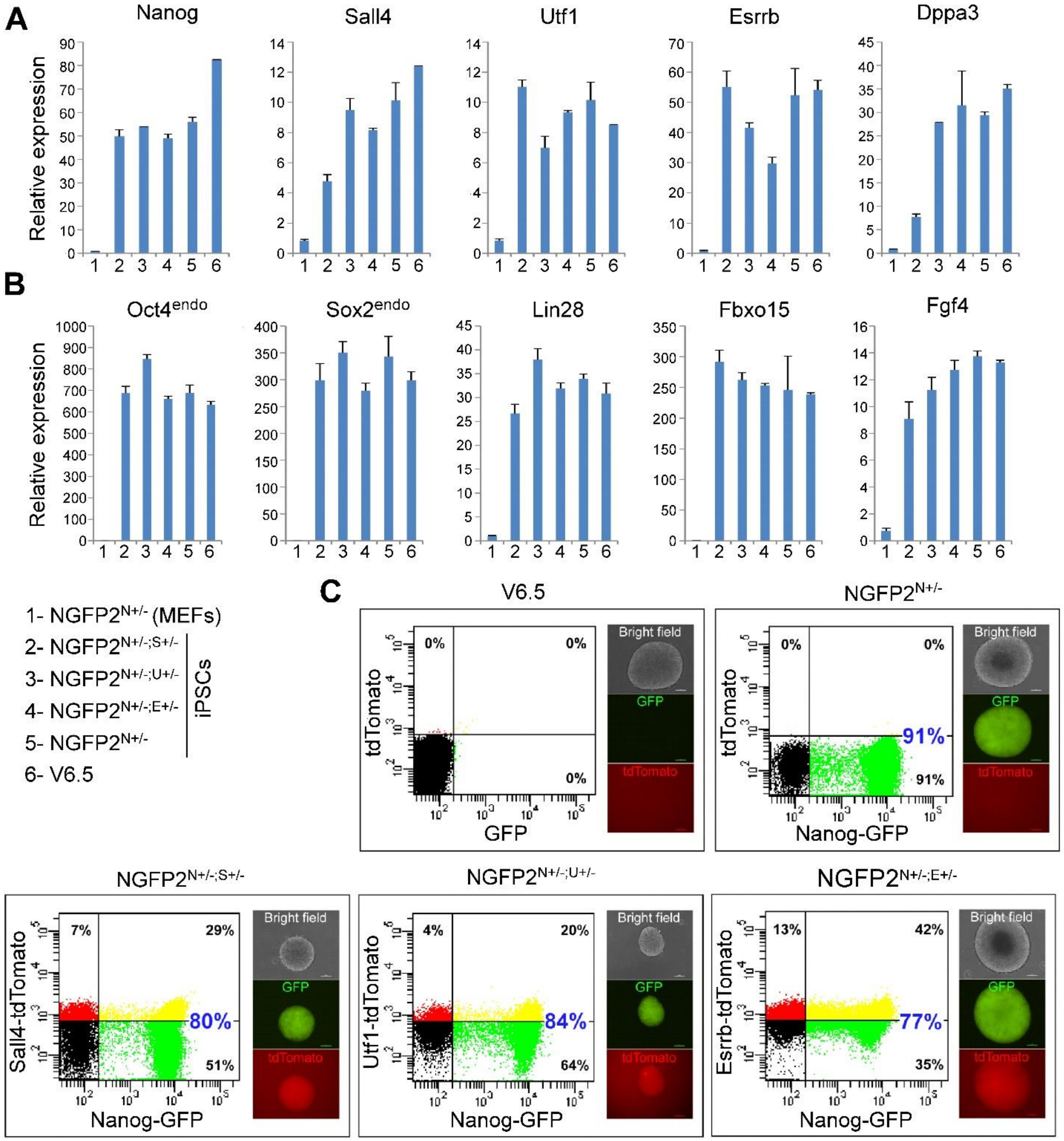

Figure S1. Characterization of the double heterozygous mutant NGFP2$^{N+/-}$ lines, related to main Figure 1. (A, B) qPCR of the indicated genes normalized to the housekeeping control gene *Gapdh* in the various NGFP2$^{N+/-}$ double heterozygous mutant lines, NGFP2$^{N+/-}$ parental line, and ESC (V6.5) and MEF. Error bars presented as a mean ± SD of 2 duplicate runs from a typical experiment out of 3 independent experiments (n= 3). **(C)** Flow cytometry analysis for GFP (*Nanog*) and tdTomato (*Utf1, Esrrb* or *Sall4*) in the various double heterozygous mutant lines that grew under 2i/L conditions. Note that the weak signal of tdTomato is due to the lack of polyA. Representative flow cytometry plots are shown out of three independent runs (n=3).

Figure S2

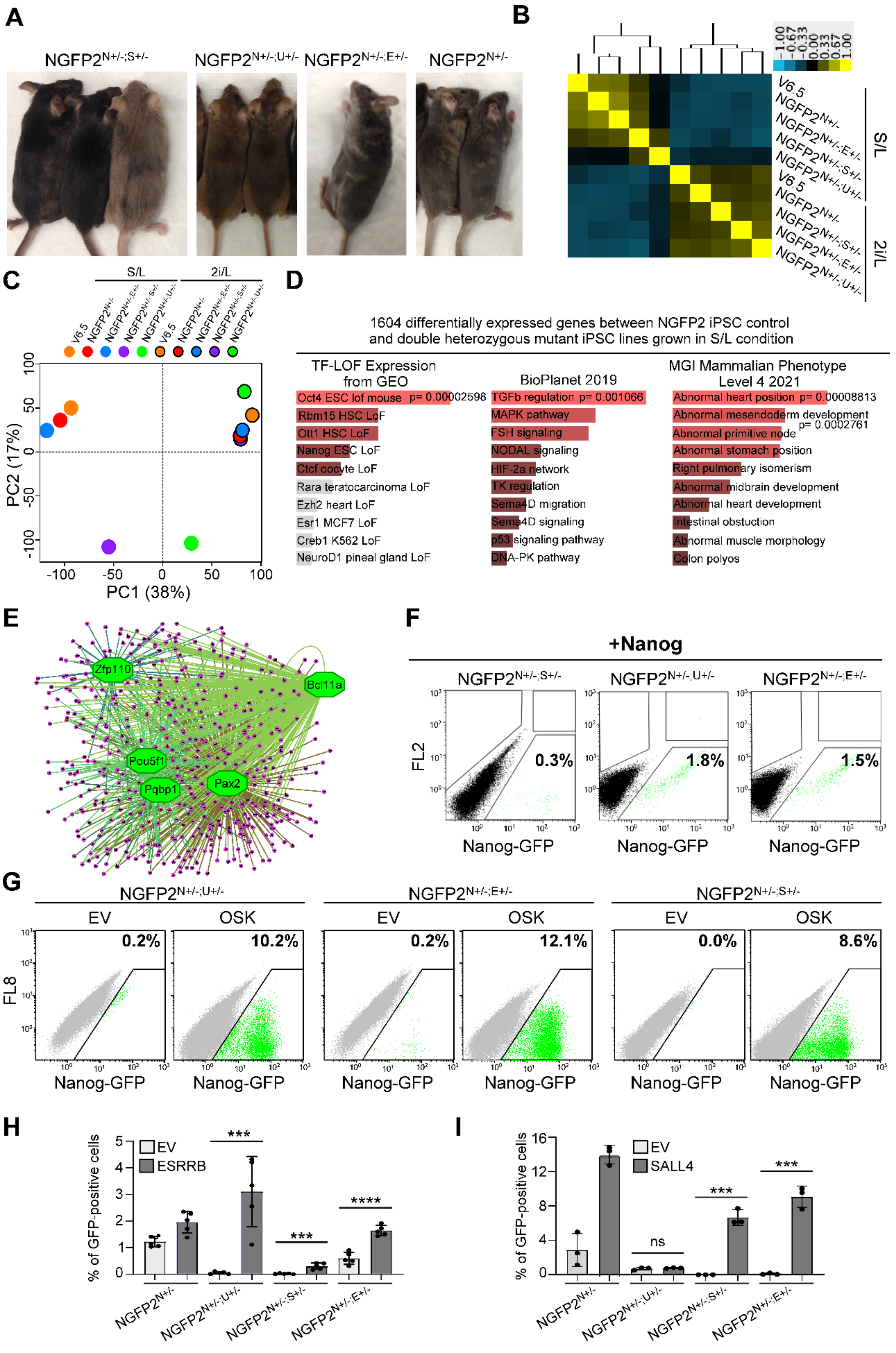

A
NGFP2N+/-;S+/-
NGFP2N+/-;U+/-
NGFP2N+/-;E+/-
NGFP2N+/-
B
-1.00 -0.67 -0.33 0.00 0.33 0.67 1.00
V6.5
NGFP2N+/-
NGFP2N+/-;E+/-
NGFP2N+/-;S+/-
NGFP2N+/-;U+/-
S/L
2i/L
C
S/L
2i/L
PC2 (17%)
PC1 (38%)
D
1604 differentially expressed genes between NGFP2 iPSC control and double heterozygous mutant iPSC lines grown in S/L condition
TF-LOF Expression from GEO
Oct4 ESC lof mouse p= 0.000002598
Rbm15 HSC LoF
Ott1 HSC LoF
Nanog ESC LoF
Ctcf oocyte LoF
Rara teratocarcinoma LoF
Ezh2 heart LoF
Esr1 MCF7 LoF
Creb1 K562 LoF
NeuroD1 pineal gland LoF
BioPlanet 2019
TGFb regulation p= 0.001066
MAPK pathway
FSH signaling
NODAL signaling
HIF-2a network
TK regulation
Sema4D migration
Sema4D signaling
p53 signaling pathway
DNA-PK pathway
MGI Mammalian Phenotype Level 4 2021
Abnormal heart position p= 0.00008813
Abnormal mesendoderm development p= 0.0002761
Abnormal primitive node
Abnormal stomach position
Right pulmonary isomerism
Abnormal midbrain development
Abnormal heart development
Intestinal obstuction
Abnormal muscle morphology
Colon polyos
E
Zfp110
Bcl11a
Pou5f1
Pqbp1
Pax2
F
+Nanog
NGFP2N+/-;S+/-
NGFP2N+/-;U+/-
NGFP2N+/-;E+/-
FL2
0.3%
1.8%
1.5%
Nanog-GFP
G
NGFP2N+/-;U+/-
NGFP2N+/-;E+/-
NGFP2N+/-;S+/-
EV
OSK
FL8
0.2%
10.2%
0.2%
12.1%
0.0%
8.6%
Nanog-GFP
H
% of GFP-positive cells
EV
ESRRB
***
***
****
NGFP2N+/-
NGFP2N+/-;U+/-
NGFP2N+/-;S+/-
NGFP2N+/-;E+/-
I
% of GFP-positive cells
EV
SALL4
ns
***
***
NGFP2N+/-
NGFP2N+/-;U+/-
NGFP2N+/-;S+/-
NGFP2N+/-;E+/-

Figure S2. The developmental potential and transcriptional profile of NGFP2$^{N+/-}$ double heterozygous mutant lines and rescue reprogramming experiments, related to main Figure 2. (A) Representative images of adult chimeric mice produced by the various NGFP2$^{N+/-}$ double heterozygous mutant iPSC lines and control following blastocyst injection and transplantation into foster mothers. For each line, 30 injected blastocysts were transferred into pseudopregnant females and born mice were analyzed. Representative images show adult chimeric mice for each line and the grade of chimerism. **(B)** Pearson Correlation heatmap and dendrogram of global gene expression profiles for two RNA-seq replicates (n=2) for the indicated NGFP2$^{N+/-}$ iPSC lines and ESC (V6.5) control grown under S/L or 2i/L conditions. Replicate pairs are assigned a shared numerical value. **(C)** Principle component analysis for the indicated samples using 500 most differentially expressed genes among all samples. Two replicates are analyzed for each sample (n=2). PC1, 38%; PC2, 17%. Each line is marked by a specific color. The group names correspond to the names in (B). Cells that were grown in 2i/L are surrounded with black circle. **(D)** Bar graphs show the most enriched GO terms and their p-value, for 1604 genes that demonstrated differential expression between ESC (V6.5)/iPSC (NGFP2$^{N+/-}$) control cells and all double heterozygous mutant iPSC lines, under S/L condition (Table S1), using EnrichR. p-value was calculated using Fisher exact test. **(E)** Gene regulatory network of the 1604 genes from (Table S1) constructed by iRegulon plugin tool in Cytoscape. Transcription factor (FDR < 0.001), Network Enrichment Score (NES) > 3. Green represents key regulators and pink marks regulated genes. Genes with no association were removed from the graph. **(F, G)** Flow cytometry analysis of Nanog-GFP-positive cells for the various NGFP2$^{N+/-}$ double heterozygous mutant induced cells following overexpression of *Nanog* (F) or OSK (G). Reprogramming occurred for 13 days with dox, followed by a 3-day dox removal. OSK indicates *Oct4*, *Sox2* and *Klf4* and EV indicates empty vector. Representative flow cytometry plots are shown out of 3 independent reprogramming runs (n=3). **(H)** Graph shows the percentage of Nanog-GFP-positive cells in the induced cells after 13 days of dox induction and 3 days of dox removal, expressing either empty vector (EV) control or ESRRB. Error bars indicate standard deviation between 5 independent experiments/replicates (n=5). ****p-value< 00001, ***p-value= 0.0009 for Utf1$^{+/-}$, and 0.0006 for Sall4$^{+/-}$ using 2-tailed unpaired t test calculated by GraphPad Prism (8.3.0). **(I)** Graph shows Nanog-GFP-positive cell percentages in the indicated induced cells after 13 days of dox induction and 3 days of dox removal, expressing either empty vector (EV) control or SALL4. Error bars indicate standard deviation between 3 independent experiments/replicates (n=3). ***p-value= 0.0002 for Sall4$^{+/-}$, and 0.0002 for Esrrb$^{+/-}$ using 2-tailed unpaired t test calculated by GraphPad Prism (8.3.0).

Figure S3

Differentially-expressed genes between control cells and all double heterozygous mutant lines at day 6 of reprogramming

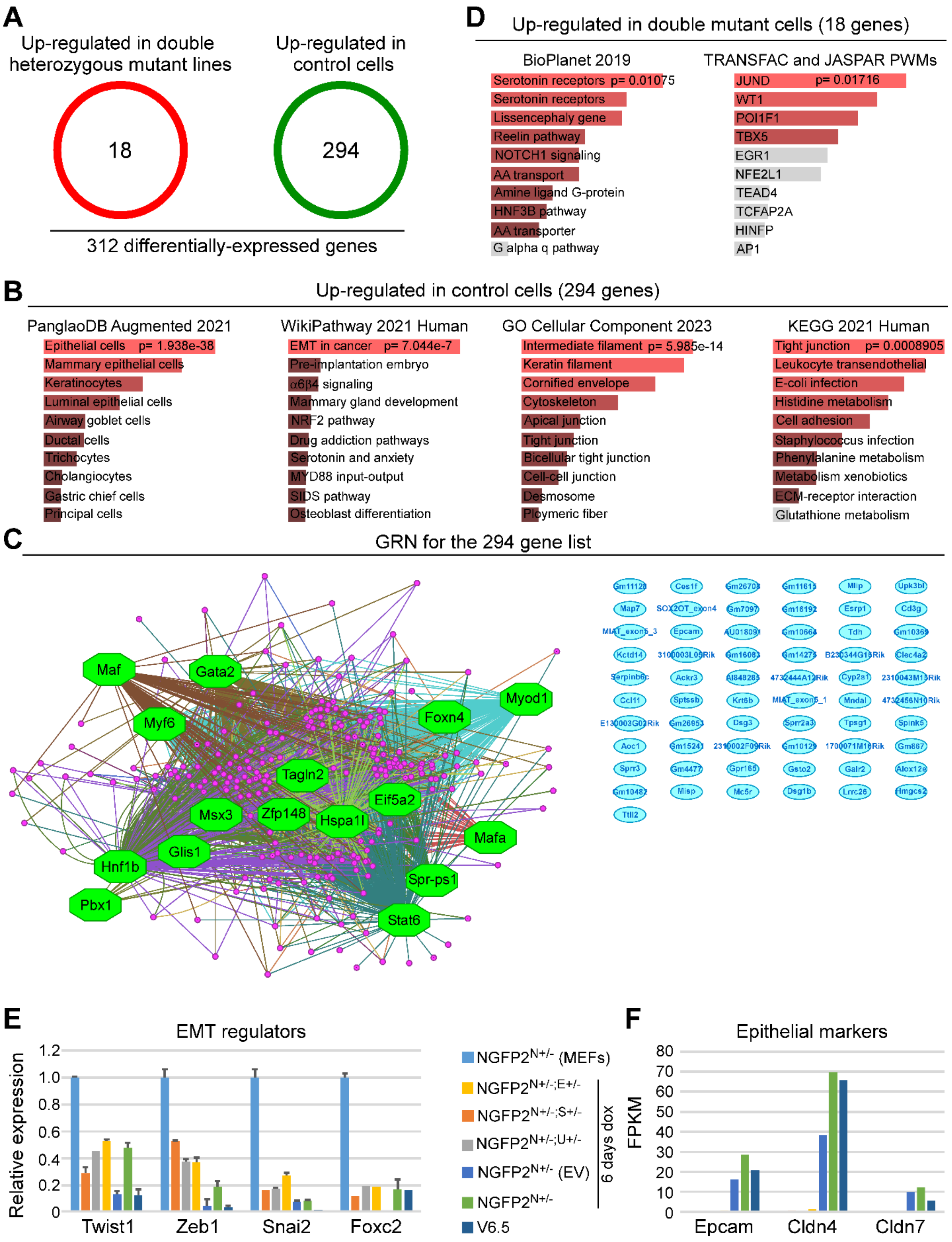

Figure S3. NGFP2$^{N+/-}$ double heterozygous mutant lines fail to activate the epithelial program during reprogramming, related to main Figure 3. (A) Schematic illustration of RNA-seq analysis depicting 18 upregulated genes in NGFP2 double heterozygous mutant lines and 294 upregulated genes in NGFP2$^{N+/-}$ control cells out of 312 differentially expressed genes (p-value< 0.05). **(B)** Bar graphs display the most enriched GO terms and their corresponding p-values for the 294 genes from (A) using EnrichR. The p-values were calculated using Fisher's exact test. **(C)** Gene regulatory network of the 294 genes from (A) constructed by iRegulon plugin tool in Cytoscape. Transcription factor (FDR < 0.001), Network Enrichment Score (NES) > 3. Green represents key regulators, pink marks regulated genes and turquoise depicts genes with no association. **(D)** Bar graphs display the most enriched GO terms and their p-value, for the 18 genes from (A) using EnrichR. p-values were calculated using Fisher exact test. **(E)** qPCR of the indicated EMT genes normalized to housekeeping control gene *Gapdh* in the various NGFP2$^{N+/-}$ double heterozygous mutant induced cells following 6 days of dox and in ESCs (V6.5) and NGFP2$^{N+/-}$ MEF control. Error bars presented as a mean ± SD of 2 duplicate runs (n=2) from a typical experiment out of 3 independent experiments (n=3). **(F)** Graph summarizes the expression level (FPKM- Fragments Per Kilobase Million) of the indicated epithelial genes in the various NGFP2$^{N+/-}$ double heterozygous mutant induced cells after 6 days of dox and in ESCs (V6.5) and NGFP2$^{N+/-}$ MEF control. Expression level of the depicted genes was obtained from the RNA-seq data described in Figure 3.

Figure S4

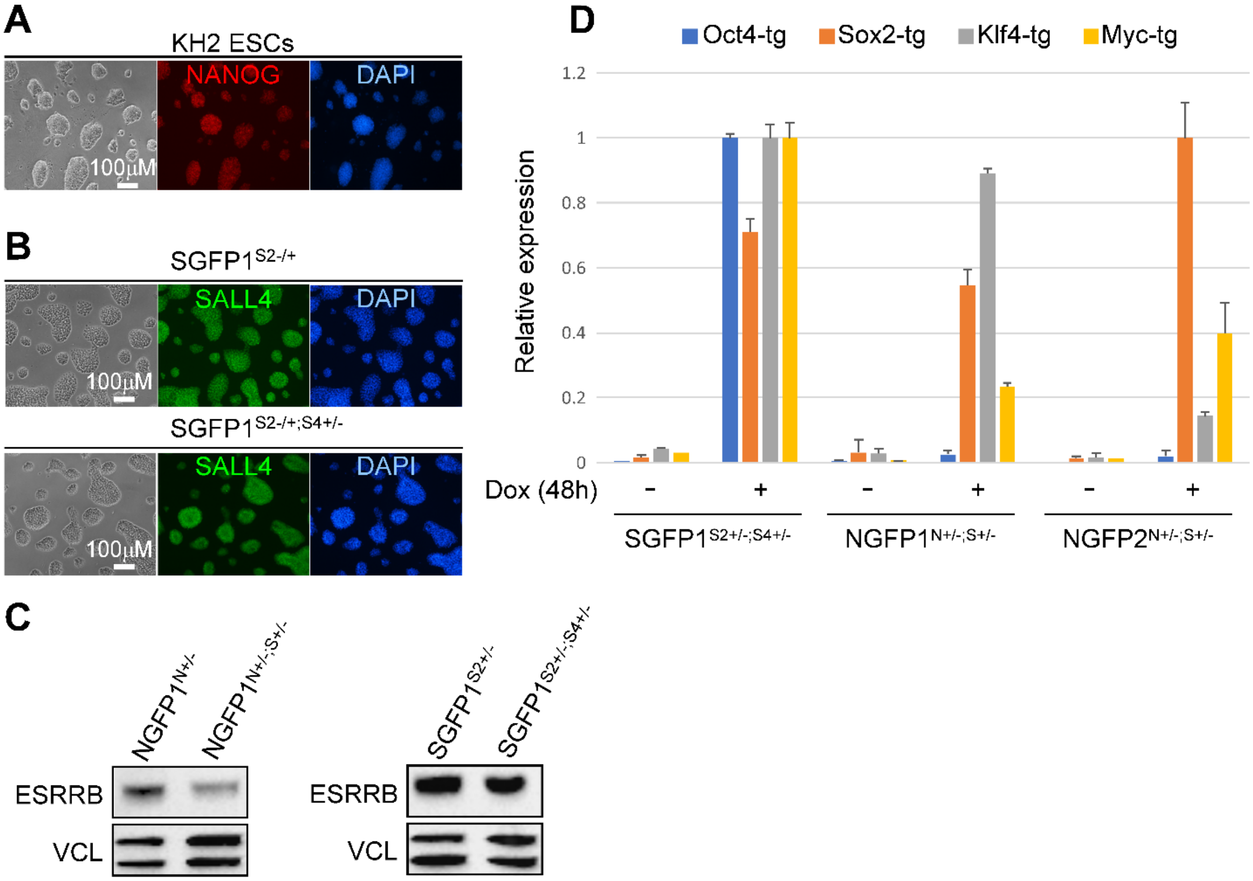
A
KH2 ESCs
NANOG
DAPI
100µM
B
SGFP1S2-/+
SALL4
DAPI
100µM
SGFP1S2-/+;S4+/-
SALL4
DAPI
100µM
C
NGFP1N+/-
NGFP1N+/-;S+/-
SGFP1S2+/-
SGFP1S2+/-;S4+/-
ESRRB
VCL
ESRRB
VCL
D
Oct4-tg
Sox2-tg
Klf4-tg
Myc-tg
Relative expression
1.2
1
0.8
0.6
0.4
0.2
0
Dox (48h)
-
+
-
+
-
+
SGFP1S2+/-;S4+/-
NGFP1N+/-;S+/-
NGFP2N+/-;S+/-

Figure S4. NANOG, SALL4 and ESRRB protein level in targeted iPSC lines and controls, related to main Figure 4. (A) Bright field and immunostaining images for NANOG (red) and DAPI (blue) in KH2 ESCs. **(B)** Bright field and immunostaining images for SALL4 (green) and DAPI (blue) in $SGFP1^{S2+/-}$ and $SGFP1^{S2+/-; S4+/-}$ iPSC lines. **(C)** Western blot analysis of the protein levels of ESRRB in $NGFP1^{N+/-;S+/-}$ and $SGFP1^{S2+/-; S4+/-}$ double heterozygous mutant iPSC lines and in their parental control cells. Cells were grown in 2i/L condition to facilitate expression from both alleles. Vinculin (VCL) was used for loading control. **(D)** qPCR of the indicated OSKM transgenes normalized to housekeeping control gene *Gapdh* in the various double heterozygous mutant MEF lines following 2 days of culture with or without dox. Error bars presented as a mean ± SD of 2 duplicate runs from a typical experiment out of three independent experiments (n=3).

Figure S5

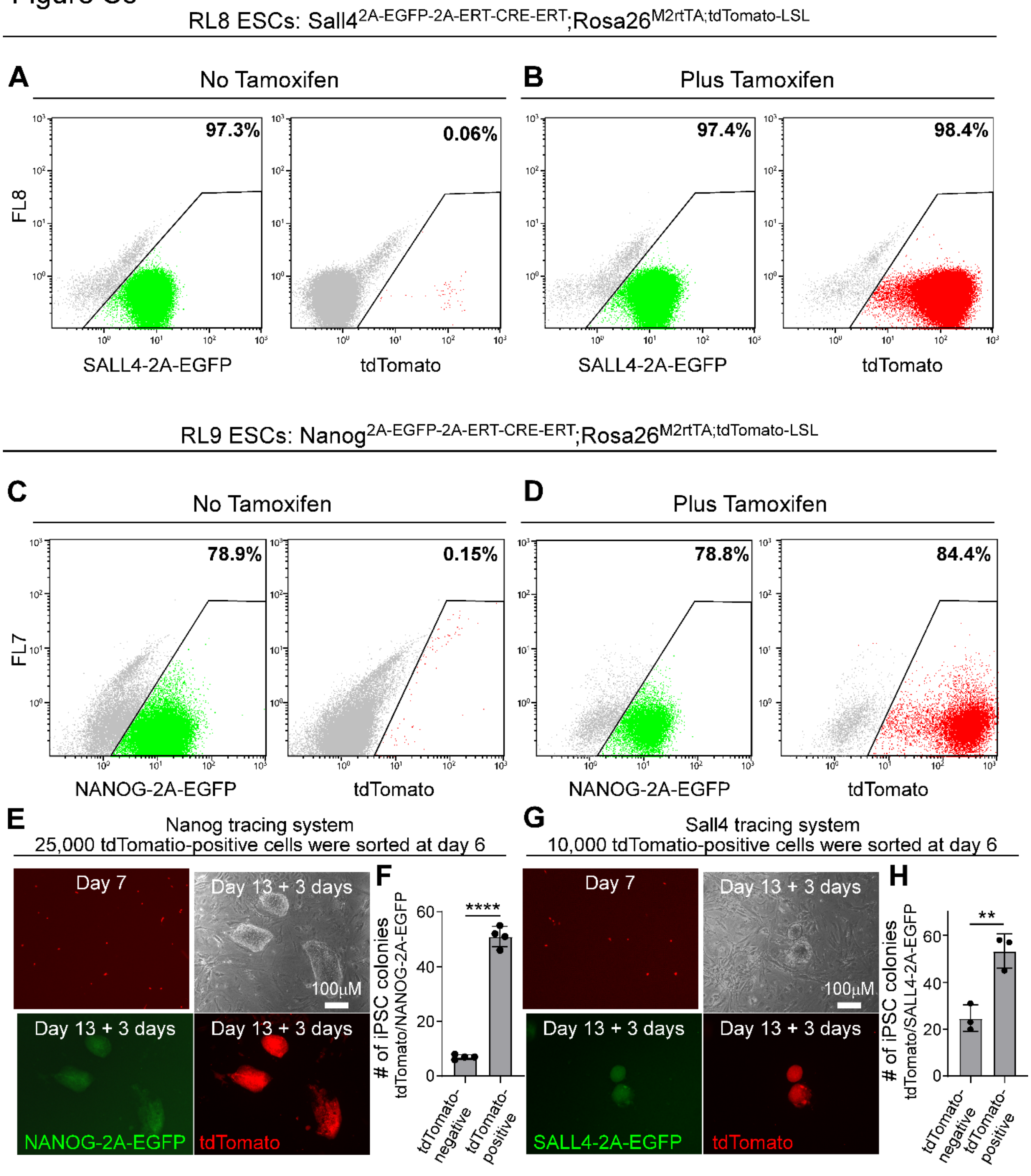
RL8 ESCs: Sall4^2A-EGFP-2A-ERT-CRE-ERT;Rosa26^M2rtTA;tdTomato-LSL
A
No Tamoxifen
B
Plus Tamoxifen
FL8
97.3%
0.06%
97.4%
98.4%
SALL4-2A-EGFP
tdTomato
SALL4-2A-EGFP
tdTomato
RL9 ESCs: Nanog^2A-EGFP-2A-ERT-CRE-ERT;Rosa26^M2rtTA;tdTomato-LSL
C
No Tamoxifen
D
Plus Tamoxifen
FL7
78.9%
0.15%
78.8%
84.4%
NANOG-2A-EGFP
tdTomato
NANOG-2A-EGFP
tdTomato
E
Nanog tracing system
25,000 tdTomatio-positive cells were sorted at day 6
Day 7
Day 13 + 3 days
100μM
Day 13 + 3 days
Day 13 + 3 days
NANOG-2A-EGFP
tdTomato
F
# of iPSC colonies
tdTomato/NANOG-2A-EGFP
****
tdTomato-negative
tdTomato-positive
G
Sall4 tracing system
10,000 tdTomatio-positive cells were sorted at day 6
Day 7
Day 13 + 3 days
100μM
Day 13 + 3 days
Day 13 + 3 days
SALL4-2A-EGFP
tdTomato
H
# of iPSC colonies
tdTomato/SALL4-2A-EGFP
**
tdTomato-negative
tdTomato-positive

Figure S5. Sall4 and Nanog tracing system characterization, related to main Figure 5. (A, B) Flow cytometry analysis for SALL4-2A-EGFP and tdTomato in the targeted ESC clone RL8 before (A) and after tamoxifen addition (B) (48 hours). Representative flow cytometry plots are out of three independent runs (n=3). **(C, D)** Flow cytometry analysis for NANOG-2A-EGFP and tdTomato in the targeted ESC clone RL9 before (C) and after tamoxifen addition (D) (48 hours). Representative flow cytometry plots are shown out of three independent runs (n=3). **(E-H)** MEFs derived from Nanog (E, F) or Sall4 (G, H) tracing system were infected with dox-inducible OSKM lentiviral vectors and reprogrammed in the presence of dox and tamoxifen for 13 days, followed by 3 days of dox removal. On day 6 of reprogramming, tdTomato-positive cells (25,000 cells for Nanog tracing system and 10,000 cells for Sall4 tracing system) were sorted and seeded on feeder-coated wells for continuous reprogramming**. (E, left upper panel)** Representative RFP channel image displays single tdTomato-positive cells from the Nanog tracing system, taken one day after sorting (Day 7). **(E, right and lower panels)** Representative bright field, RFP and green channel images of stable iPSC colonies from tdTomato-positive cells at the end of the reprogramming process. **(F)** Graph summarizes the number of tdTomato/EGFP-positive iPSC colonies generated from tdTomato-negative and tdTomato-positive sorted cells using the *Nanog* tracing system. Error bars indicate standard deviation between 4 independent experiments/replicates (n=4). ****p-value< 0.0001 using 2-tailed unpaired t test calculated by GraphPad Prism (8.3.0). **(G, left upper panel)** Representative RFP channel image shows single tdTomato-positive cells from Sall4 tracing system one day after sorting (Day 7). **(G, right and lower panels)** Representative bright field, RFP and green channel images of stable iPSC colonies from tdTomato-positives cells at the end of the reprogramming process. **(H)** Graph summarizes the number of tdTomato/EGFP-positive iPSC colonies generated from tdTomato-negative and tdTomato-positive sorted cells using the Sall4 tracing system. Error bars indicate standard deviation between 3 independent experiments/replicates (n=3). **p-value= 0.0057 using 2-tailed unpaired t test calculated by GraphPad Prism (8.3.0).

Figure S6

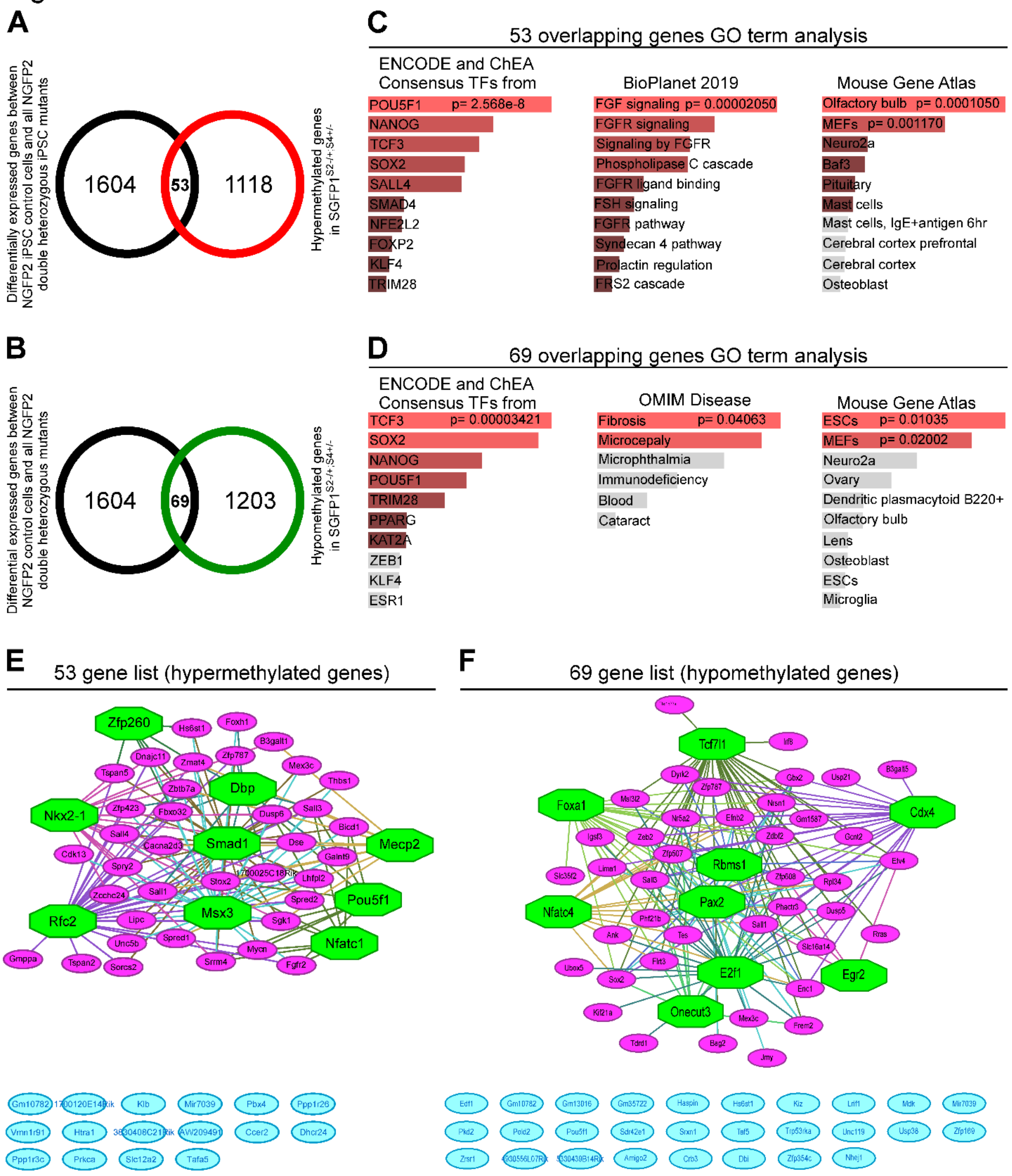
A
Differentially expressed genes between NGFP2 iPSC control cells and all NGFP2 double heterozygous iPSC mutants
1604
53
1118
Hypermethylated genes in SGFP1$^{S2-/+;S4+/-}$
B
Differential expressed genes between NGFP2 control cells and all NGFP2 double heterozygous mutants
1604
69
1203
Hypomethylated genes in SGFP1$^{S2-/+;S4+/-}$
C
53 overlapping genes GO term analysis
ENCODE and ChEA Consensus TFs from
POU5F1 p= 2.568e-8
NANOG
TCF3
SOX2
SALL4
SMAD4
NFE2L2
FOXP2
KLF4
TRIM28
BioPlanet 2019
FGF signaling p= 0.00002050
FGFR signaling
Signaling by FGFR
Phospholipase C cascade
FGFR ligand binding
FSH signaling
FGFR pathway
Syndecan 4 pathway
Prolactin regulation
FRS2 cascade
Mouse Gene Atlas
Olfactory bulb p= 0.0001050
MEFs p= 0.001170
Neuro2a
Baf3
Pituitary
Mast cells
Mast cells, IgE+antigen 6hr
Cerebral cortex prefrontal
Cerebral cortex
Osteoblast
D
69 overlapping genes GO term analysis
ENCODE and ChEA Consensus TFs from
TCF3 p= 0.00003421
SOX2
NANOG
POU5F1
TRIM28
PPARG
KAT2A
ZEB1
KLF4
ESR1
OMIM Disease
Fibrosis p= 0.04063
Microcepaly
Microphthalmia
Immunodeficiency
Blood
Cataract
Mouse Gene Atlas
ESCs p= 0.01035
MEFs p= 0.02002
Neuro2a
Ovary
Dendritic plasmacytoid B220+
Olfactory bulb
Lens
Osteoblast
ESCs
Microglia
E
53 gene list (hypermethylated genes)
Zfp260
Nkx2-1
Dbp
Smad1
Mecp2
Pou5f1
Msx3
Nfatc1
Rfc2
Hs6st1
Foxh1
B3galt1
Dnajc11
Zfp787
Zmat4
Tspan5
Mex3c
Thbs1
Zbtb7a
Zfp423
Fbxo32
Dusp6
Sall3
Sall4
Cacna2d3
Bicd1
Dse
Cdk13
Galnt9
Spry2
1700025C18Rik
Lhfpl2
Stox2
Zcchc24
Sall1
Spred2
Lipc
Sgk1
Unc5b
Spred1
Mycn
Gmppa
Tspan2
Sorcs2
Srrm4
Fgfr2
Gm10782
1700120E14Rik
Klb
Mir7039
Pbx4
Ppp1r26
Vmn1r91
Htra1
3830408C21Rik
AW209491
Ccer2
Dhcr24
Ppp1r3c
Prkca
Slc12a2
Tafa5
F
69 gene list (hypomethylated genes)
Tcf7l1
Foxa1
Cdx4
Rbms1
Pax2
Nfatc4
E2f1
Egr2
Onecut3

Figure S6. The specific transcriptome of NGFP2$^{N+/-}$ double heterozygous mutant iPSCs exhibit similarities to SGFP1$^{S2+/-}$ double heterozygous MEF methylation profile, related to main Figure 6. **(A)** Venn diagram displays the 53 overlapping genes (p-value< 0.00001, Fisher exact test) between the 1604 differentially expressed genes in NGFP2$^{N+/-}$ iPSC control versus all NGFP2 double heterozygous mutant iPSC lines and the 1118 hypermethylated genes in SGFP1$^{S2+/-;S4+/-}$ MEFs versus SGFP1$^{S2+/-}$ control MEFs. **(B)** Venn diagram shows the 69 overlapping genes (p-value< 0.00001, Fisher exact test) between the 1604 differentially expressed genes in NGFP2$^{N+/-}$ iPSC control versus all NGFP2 double heterozygous mutant iPSC lines and the 1203 hypormethylated genes in SGFP1$^{S2+/-;S4+/-}$ MEFs versus SGFP1$^{S2+/-}$ control MEFs. **(C, D)** Bar graphs display the most enriched GO terms and their p-value, for the 53 or 69 genes from (A) and (B), respectively using EnrichR. p-values were calculated using Fisher exact test. **(E,F)** Gene regulatory network of the 53 overlapping genes (E) or 69 overlapping genes (F) from (A) and (B), respectively constructed by iRegulon plugin tool in Cytoscape. Transcription factor (FDR < 0.001), Network Enrichment Score (NES) > 3. Green represents key regulators, pink marks regulated genes and turquoise depicts genes with no association.

EXPERIMENTAL PROCEDURES

Cell culture

Mouse embryonic fibroblasts (MEFs) were isolated as previously described (Wernig et al., 2008). MEFs were grown in DMEM supplemented with 10% fetal bovine serum, 1% non-essential amino acids, 2 mM L-Glutamine and antibiotics. ESCs and iPSCs were grown in S/L medium or 2i/L: DMEM supplemented with 10% fetal bovine serum, 1% non-essential amino acids, 2 mM L-Glutamine, 2X106 units mLif, 0.1 mM β-mercaptoethanol (Sigma) and antibiotics with or without 2i- PD0325901 (1 mM) and CHIR99021 (3 mM) (PeproTech). All the cells were maintained in a humidified incubator at 37°C and 6% CO2. All infections were performed on MEFs (passage 0-2) that were seeded at 50-70% confluency two days before the first infection. During the reprogramming to iPSC, the cells were grown in S/L medium with the addition of 2 μg/ml doxycycline.

Secondary MEF production

Briefly, iPSC lines (NGFP2, NGFP1 and SGFP1 lines) were injected into blastocysts and chimeric embryos were isolated at E13.5. For MEF production, embryos were dissected under the binocular removing internal organs and heads. The remaining body was chopped thoroughly by scalpels and exposed to 1ml Tripsin-EDTA (0.25%, GIBCO) for 30 minutes at 37°C. Following that, 10 mL of DMEM medium containing 10%FBS was added to the plate and the chopped tissue was subjected to thorough and intensive pipetting resulting in a relatively homogeneous mix of cells. Each chopped embryo was seeded in 15cm plate and cells were cultured in DMEM supplemented with 10%FBS, 2mM L-glutamine, and antibiotics until the plate was full. Puromycin (2 μg/ml) was added to each 15cm plate for positive selection for NGFP2, NGFP1 and SGFP1 MEFs, eliminating only the host cells.

Immunostaining and Western blot

Cells were fixed in 4% paraformaldehyde (in PBS) for 20 minutes. The cells were rinsed 3 times with PBS and blocked for 1hr with PBS containing 0.1% Triton X-100 and 5% FBS. The cells were incubated overnight with primary antibodies (1:200) in 4C. The antibodies are: anti-SALL4 (Abcam, ab29112, 1:500) and anti-NANOG (Bethyl, A300-379A, 1:500) in PBS containing 0.1% Triton X-100 and 1%FBS. The next day, the cells were washed 3 times and incubated for 1hr with relevant (Alexa, 1:500) secondary antibody in PBS containing 0.1% Triton X-100 and 1% FBS. DAPI (1:1000 dilution) was added 10 minutes before the end of incubation. For western blot, cell pellets were lysed on ice in lysis buffer (20 mM Tris-HCl, pH 8, 1mM EDTA pH 8, 0.5% Nonidet P-40, 150mM NaCl, 10% glycerol, 1mM, protease inhibitors (Roche Diagnostics)

for 10 min, supernatant were collected and 40μg protein were suspended with sample buffer and boiled for or 5 min at 100C, and subjected to western blot analysis. Primary antibodies: anti-SALL4 (Abcam, ab29112, 1:500), anti-NANOG (Bethyl, A300-379A, 1:500), anti-ESRRB (Perseus proteomics, PP-H6705-00, 1:500), anti-UTF1 (Abcam, ab24273, 1:500), anti-ACTB (Santa cruz, sc-1616, 1:500), anti-β-TUBULIN (Abcam, ab179513, 1:500), anti-VCT (Abcam, ab129002, 1:500). Blots were probed with anti-mouse, anti-goat or anti-rabbit IgG-HRP secondary antibody (1:10,000) and visualized using ECL detection kit.

Quantitative real-time PCR

Total RNA was isolated using the Macherey-Nagel kit (Ornat). 500–2000 ng of total RNA was reverse transcribed using iScript cDNA Synthesis kit (Bio-Rad). Quantitative PCR analysis was performed in duplicates using 1/100 of the reverse transcription reaction in a StepOnePlus (Applied Biosystems) with SYBR green Fast qPCR Mix (Applied Biosystems). Specific primers flanking an intron were designed for the different genes (see Primer Table S2). All quantitative real-time PCR experiments were repeated at least three times, and the results were normalized to the expression of *Gapdh* and presented as a mean ± standard deviation of two duplicate runs from a typical experiment.

Southern Blot

Southern blot was performed as previously described (Carey et al., 2011). For primer list see Table S2.

FACS analysis

Cells were washed twice with PBS and trypsinized (0.25%) and filtered through mesh paper. Flow cytometry analysis was performed on a Beckman Coulter and analyzed using Kaluza Software. All FACS experiments were repeated at least three times, and the bar graph results are presented as a mean ± standard deviation of two biological duplicate from a typical experiment. Flow cytometry analysis was performed on a Beckman Coulter and analyzed using Kaluza Software.

RNA sequencing

Total RNA was isolated using Rneasy Kit (QIAGEN) and sent to the “Technion Genome Center”, Israel, for library preparation and sequencing.

Cleaning and filtering of raw reads

Raw reads (fastq files) were inspected for quality issues with FastQC (v0.11.2, http://www.bioinformatics.babraham.ac.uk/projects/fastqc/). According to the FastQC report, reads were then trimmed to a length of 50 bases with fastx_trimmer of the FASTX package (version 0.0.13, http://hannonlab.cshl.edu/fastx_toolkit/), and quality-trimmed at both ends, using in-house perl scripts, with a quality threshold of 32. In short, the scripts use a sliding window of 5 base pairs from the read's end and trim one base at a time until the average quality of the window passes the given threshold. Following quality-trimming, adapter sequences were removed by Trim Galore (version 0.3.7, http://www.bioinformatics.babraham.ac.uk/projects/trim_galore/), using the command "trim_galore -a $adseq –length 15" where $adseq is the appropriate adapter sequence. The remaining reads were further filtered to remove very low-quality reads, using the fastq_quality_filter program of the FASTX package, with a quality threshold of 20 at 90 percent or more of the read's positions.

Expression analysis

The cleaned fastq files were mapped to the mouse transcriptome and genome, Ensembl version GRCm38 from Illumina's iGenomes (http://support.illumina.com/sequencing/sequencing_software/igenome.html), using TopHat (v2.0.11), allowing up to 3 mismatches and a total edit distance of 8 (full command: tophat -G Mus_musculus/Ensembl/GRCm38/Annotation/Genes/genes.gtf -N 3 --read-gap-length 5 --read-edit-dist 8 --segment-length 18 --read-realign-edit-dist 5 --b2-i S,1,0.75 --b2-mp 3,1 --b2-score-min L,-0.5,-0.5 Mus_musculus/Ensembl/GRCm38/Sequence/Bowtie2Index/genome clean.fastq). Quantification and normalization were done with the Cufflinks package (v2.2.1). Quantification was done with cuffquant, using the genome bias correction (-b parameter), multi-mapped reads assignment algorithm (-u parameter) and masking for genes of type IG, TR, pseudo, rRNA, tRNA, miRNA, snRNA and snoRNA (-M parameter). Normalization was done with cuffnorm (using output format of Cuffdiff).

Visualization

The R package cummeRbund (version 2.8.2) was used to calculate and draw the figures (such as scatter plots, MA plots, etc.) from the normalized expression values.

Chimera Formation

Blastocyst injections were performed using (C57/Bl6xDBA) B6D2F2 or CB6F1 host embryos. All injected iPSC lines were derived from crosses of 129Sv/Jae to C57/Bl6 mice and could be identified by agouti coat color. Embryos were obtained 24 hr (1 cell stage) or 40 hr (2 cell stage) posthuman chorionic gonadotropin (hCG) hormone priming. Diploid embryos were cultured in EmbryoMax KSOM (Millipore) or Evolve KSOMaa (Zenith Biotech) until they formed blastocysts (94–98 hr after hCG injection) at which point they were placed in a drop of Evolve w/HEPES KSOMaa (Zenith) medium under mineral oil. A flat tip microinjection pipette with an internal diameter of 16 mm (Origio) was used for iPSC injections. Each blastocyst received 8–12 iPSCs. Shortly after injection, blastocysts were transferred to day 2.5 recipient CD1 females (20 blastocysts per female). Pups, when not born naturally, were recovered at day 19.5 by cesarean section and fostered to lactating Balb/c mothers.

Nuclear transfer

Nuclear transfer was performed as described (Wakayama et al., 1998) with modifications. Briefly, metaphase II-arrested oocytes were collected from superovulated B6D2F1 females (8-10 wks) and cumulus cells were removed using hyaluronidase. The oocytes were enucleated in a droplet of HEPES-CZB medium containing 5μg/ml cytochalasin B (CB) using a blunt Piezo-driven pipette. After enucleation, the spindle-free oocytes were washed extensively and maintained in CZB medium up to 2 h before nucleus injection. The CCs from mice (B6D2F1) were aspirated in and out of the injection pipette to remove the cytoplasmic material and then injected into enucleated oocytes. The reconstructed oocytes were cultured in CZB medium for 1 h and then activated for 5-6 h in activation medium containing 10mM Sr 2+, 5ng/ml trichostatin A (TSA) and 5μg/ml CB. Following activation, all of the re constructed embryos were cultured in KSOM medium supplemented with 5ng/ml TSA for another 3-4 hours and maintained in KSOM medium with amino acids at 37C under 5% CO2 in air.

Reduced-representation bisulfite sequencing (RRBS)

DNA was isolated from MEFs and incubated in lysis buffer (25 mM Tris-HCl (pH 8), 2 mM ethylenediaminetetraacetic acid, 0.2% sodium dodecyl sulfate, 200 mM NaCl) supplemented with 300 μg/mL proteinase K (Roche) followed by phenol:chloroform extraction and ethanol precipitation and RRBS libraries were prepared (Boyle et al., 2012) and run on HiSeq 2500 (Illumina) using 100 bp paired-end sequencing.

DNA methylation was analyzed by using 100 bp paired-end sequencing reads from RRBS that were trimmed and quality filtered by trim galore software using default parameters for RRBS. Read alignment (genome build mm10) and extraction of single-base resolution methylation levels were carried out by BSMAP. Differentially methylated regions (DMR) were explored with R methylKit package version 1.18.0 (Akalin et al., 2012). CpG sites featuring less than 10 reads were considered unreliable and discarded from further analysis. CpG sites were then aggregated into consecutive tiles of size 100 bp and a threshold of at least 15 reads per tile was applied. Differential methylation between the two lines, each consisting of three samples, was determined by logistic regression and adjusted p-values are calculated with SLIM (sliding linear model). A threshold of 1E-3 was set for adjusted p-value and a threshold of 20 methylation points was set between the two lines and further explored. DMRs were annotated with Homer (Hypergeometric Optimization of Motif Enrichment) version 4.11.1 (Heinz et al., 2010) and specifically its function annotatePeaks.pl. This function outputs a set of genes affiliated with DMR based on the nearest promoter distance. Heatmaps were created with R package heatmap.2 version 3.1.1 and dendrogram with R package dendextend version 1.15.2 (Galili, 2015).

Figure legends and tables

Table S1. Differential expressed genes and genomic loci between control and double heterozygous mutant lines, related to main Figures 3, 6, and Supplementary Figures S2, S3, S6.

Table S2. primer list used in this study, related to main Figures 3, 4, 5, and Supplementary Figures S1, S3, S4.

Gene	Application	Primer Sequence (5' --> 3')
Gapdh	qPCR analysis of mRNA expression normalization	F: CCTCAACGACCACTTTGTCAAG<br>R: TCTTCCTCTTGTGCTCTTGCTG
Thy1	qPCR analysis of mRNA expression	F: CCAGAACGTCACAGTGCTCA<br>R: AGGTGTTCTGAGCCAGCAG
Col5a2	qPCR analysis of mRNA expression	F: TAGAGGAAGAAAGGGACAAAAAGG<br>R: GTTACAACAGGCACTAATCCTGGTT
Postn	qPCR analysis of mRNA expression	F: ACAACAATCTGGGGCTTTTT<br>R: AATCTGGTTCCCATGGATGA
Des	qPCR analysis of mRNA expression	F: TGGAGCGTGACAACCTGATA<br>R:AAGGCAGCCAAGTTGTTCTC
Cdh1	qPCR analysis of mRNA expression	F: CTCGACACCCGATTCAAAGT<br>R: GGCGTAGACCAAGAAATGGA

Dsp	qPCR analysis of mRNA expression	F: ACCGTCAACGACCAGAACTC<br>R: TTTGCAGCATTTCTTGGATG
Nanog	qPCR analysis of mRNA expression	F: AAACCAGTGGTTGAAGACTAGCAA<br>R: GGTGCTGAGCCCTTCTGAATC
Oct4 endogenous	qPCR analysis of mRNA expression	F: TCAGTGATGCTGTTGATCAGG<br>R: GCTATCTACTGTGTGTCCCAGTC
Sox2 endogenous	qPCR analysis of mRNA expression	F: CCGTTTTCGTGGTCTTGTTT<br>R: TCAACCTGCATGGACATTTT
Lin28	qPCR analysis of mRNA expression	F: GAAGAACATGCAGAAGCGAAGA<br>R: CCGCAGTTGTAGCACCTGTCT
Fbxo15	qPCR analysis of mRNA expression	F: CGAGAATGGTGGACTAGCTTTTG<br>R: GGCCATGGGAATGAATATTTG
Fgf4	qPCR analysis of mRNA expression	F: GCAGACACGAGGGACAGTCT<br>R: ACTCCGAAGATGCTCACCAC
Sall4	qPCR analysis of mRNA expression	F: GCAAGTCACCAGGGCTCTT<br>R: CCTCCTTAGCTGACAGCAATC
Utf1	qPCR analysis of mRNA expression	F: GTCCCTCTCCGCGTTAGC<br>R: GGCAGGTTCGTCATTTTCC
Esrrb	qPCR analysis of mRNA expression	F: CACCTGCTAAAAAGCCATTGACT<br>R: CAACCCCTAGTAGATTCGAGACGAT
Dppa3	qPCR analysis of mRNA expression	F: TCGGATTGAGCAGAGACAAAAA<br>R: TCCCGTTCAAACTCATTTCCTT
Twist1	qPCR analysis of mRNA expression	F: ACGCTGCCCTCGGACAA<br>R: CCTGGCCGCCAGTTTG
Zeb1	qPCR analysis of mRNA expression	F: CCAGGTGTAAGCGCAGAAAG<br>R: TCATCGGAATCTGAATTTGCT
Snai2	qPCR analysis of mRNA expression	F: ATCCTCACCTCGGGAGCATA<br>R: TGCCGACGATGTCCATACAG
Foxc2	qPCR analysis of mRNA expression	F: AGAACAGCATCCGCCACAAC<br>R: GCACTTTCACGAAGCACTCATT
Oct4-transgene	qPCR analysis of transgenic mRNA expression	F : CGCCTGGAGACGCCATCCACGCT<br>R: GTTGGTTCCACCTTCTCCAA
Sox2-transgene	qPCR analysis of transgenic mRNA expression	F: GCCCAGTAGACTGCACATGG<br>R: AGAATACCAGTCAATCTTTCA
Klf4-transgene	qPCR analysis of transgenic mRNA expression	F: CGCCTGGAGACGCCATCCACGCT<br>R: ACGCAGTGTCTTCTCCCTTC
Myc-transgene	qPCR analysis of transgenic mRNA expression	F : TGTCCATTCAAGCAGACGAG<br>R: AGAATACCAGTCAATCTTTCA
Nanog gRNA	gRNA for generating Nanog KO iPSCs	F: CACCGAGAACTATTCTTGCTTACA<br>R: AAACTGTAAGCAAGAATAGTTCTC
Nanog KO	KO validation PCR	F: CGGCTCACTTCCTTCTGACT<br>R: TATTGCTCCGTCCTGTGTCC
Nanog tracing 5 arm	PCR for generating arm for targeting vector	F : TAACAGCTGAAGTACCTCAGCCTCCAGCA<br>R:TAACAGCTGTATTTCACCTGGTGGAGTCACA
Nanog tracing 3 arm	PCR for generating arm for targeting vector	F: GGTACCCCAGCCCCTGGTTTATTTTT<br>R: CCGCGGACCCACACAGCCTCTCAAGT

Nanog gRNA	gRNA tracing	F: CACCGGATTTGAACTCCTGACCTT<br>R: AAACAAGGTCAGGAGTTCAAATCC
Nanog validation 5 arm tracing	PCR analysis of integration into genomic DNA	F: CCACCCCGTGAACTGACT<br>R: CGTCACCGCATGTTAGAAGA
Nanog validation 3 arm tracing	PCR analysis of integration into genomic DNA	F : GGTACCCCAGCCCCTGGTTTATTTTT<br>R : CCCTGTGAGTGGTCAGGAGT
Sall4 tracing 5 arm	PCR for generating arm for targeting vector	F: GTTAACGCAAGGGAGAGCCAGTATT<br>R: GTTAACGCTGACAGCAATCTTATT
Sall4 tracing 3 arm	PCR for generating arm for targeting vector	F: GGTACCCTGATATGCAAGTGATGT<br>R: CCGCGGATACACACAAGCCCGCCTC
Sall4 gRNA	gRNA tracing	F: CACCGGAGGAGAGGAGTCTTCTGC<br>R: AAACGCAGAAGACTCCTCTCCTCC
Sall4 validation 5 arm tracing	PCR analysis of integration into genomic DNA	F: TAATCCAGCCTTGCTCGTCT<br>R: CGTCACCGCATGTTAGAAGA
Sall4 validation 3 arm tracing	PCR analysis of integration into genomic DNA	F: ACAGCTGTCGAGGTACCCTGA<br>R: GTGTGTGTGTGTCCGTCCTC
Nanog-cDNA	Primers used for cloning of cDNA for lentiviral gene overexpression	F: CGCCATCACACTGACATGA<br>R: TGGAAGAAGGAAGGAACCTG
Sall4-cDNA	Primers used for cloning of cDNA for lentiviral gene overexpression	F: GCAAGTCACCAGGGCTCTT<br>R: CCTCCTTAGCTGACAGCAAT
Esrrb-cDNA	Primers used for cloning of cDNA for lentiviral gene overexpression	F: GCTGGAACACCTGAGGGTAA<br>R: GGTCTCCACTTGGATCGTGT
Utf1-cDNA	Primers used for cloning of cDNA for lentiviral gene overexpression	F: CTACCTGGCTCAGGGATGCT<br>R: GACTGGGAGTCGTTTCTGGA
Sall4 gRNA	gRNA for generating Sall4 KI/KO in NGFP1 and SGFP1	F: CACCGCCAGCTCTCCGCGGATGGT<br>R: AAACACCATCCGCGGAGAGCTGGC
Sall4 5arm validation PCR	PCR analysis of integration into genomic DNA	F: CATACACAAAGCCCCAGGTT<br>R: GCGCATGAACTCTTTGATGA
Sall4 3arm validation PCR	PCR analysis of integration into genomic DNA	F: CGGGATCCGAAGTTCCTATT<br>R: AGCTTGCAAAGGGAAAGACA
Utf1 KI/KO targeting 5arm	PCR for generating arm for targeting vector	F: GAACAGGCTTTTGGCTTCAG<br>R: GGCGCTGGGGACGTCCAGGG<br>Product size: 2920 bps
Utf1 KI/KO targeting 3arm	PCR for generating arm for targeting vector	F: GGCCATACCTTCGAATCCTC<br>R: CCAACACCCAAGAGAAGAGG<br>Product size: 1905 bps
Esrrb KI/KO targeting 5arm	PCR for generating arm for targeting vector	F: AGACACAAGGCTGGAGAGGA<br>R: GGTACCGTGGTAGCCAGAGGCAATG<br>Product size : 3050 bps
Esrrb KI/KO targeting 3arm	PCR for generating arm for targeting vector	F: GGGACCTCAAGGTGAAATGA<br>R: TAAGCCCAACACCTGGAAAC<br>Product size: 3400 bps

Sall4 KI/KO targeting 5arm	PCR for generating arm for targeting vector	F: CAGCCTGGGCTACTTGAGAC<br>R: CTCCTCCCAGTTGATGTGCT<br>Product size: 3200 bps
Sall4 KI/KO targeting 3arm	PCR for generating arm for targeting vector	F: TGGTCCACCTGGAACAAAAG<br>R: AGAAGGGAGCTATGGCACAA<br>Product size: 3155 bps

REFERENCES

Akalin, A., Kormaksson, M., Li, S., Garrett-Bakelman, F.E., Figueroa, M.E., Melnick, A., and Mason, C.E. (2012). methylKit: a comprehensive R package for the analysis of genome-wide DNA methylation profiles. Genome Biol *13*, R87.

Boyle, P., Clement, K., Gu, H., Smith, Z.D., Ziller, M., Fostel, J.L., Holmes, L., Meldrim, J., Kelley, F., Gnirke, A., *et al.* (2012). Gel-free multiplexed reduced representation bisulfite sequencing for large-scale DNA methylation profiling. Genome Biol *13*, R92.

Carey, B.W., Markoulaki, S., Hanna, J.H., Faddah, D.A., Buganim, Y., Kim, J., Ganz, K., Steine, E.J., Cassady, J.P., Creyghton, M.P., *et al.* (2011). Reprogramming factor stoichiometry influences the epigenetic state and biological properties of induced pluripotent stem cells. Cell stem cell *9*, 588-598.

Galili, T. (2015). dendextend: an R package for visualizing, adjusting and comparing trees of hierarchical clustering. Bioinformatics *31*, 3718-3720.

Heinz, S., Benner, C., Spann, N., Bertolino, E., Lin, Y.C., Laslo, P., Cheng, J.X., Murre, C., Singh, H., and Glass, C.K. (2010). Simple combinations of lineage-determining transcription factors prime cis-regulatory elements required for macrophage and B cell identities. Mol Cell *38*, 576-589.

Wakayama, T., Perry, A.C., Zuccotti, M., Johnson, K.R., and Yanagimachi, R. (1998). Full-term development of mice from enucleated oocytes injected with cumulus cell nuclei. Nature *394*, 369-374.

Wernig, M., Lengner, C.J., Hanna, J., Lodato, M.A., Steine, E., Foreman, R., Staerk, J., Markoulaki, S., and Jaenisch, R. (2008). A drug-inducible transgenic system for direct reprogramming of multiple somatic cell types. Nat Biotechnol *26*, 916-924.
