# Supplementary material for: Complex haploinsufficiency in pluripotent cells yields somatic cells with DNA methylation abnormalities and pluripotency induction defects
Source: Stem Cell Reports. 2023 Oct 12;18(11):2174–89. doi: 10.1016/j.stemcr.2023.09.009 (PMC10679652; doi:10.1016/j.stemcr.2023.09.009)
Supplement: Document S2. Article plus supplemental information [file mmc3.pdf]

Stem Cell Reports
Article

OPEN ACCESS

Complex haploinsufficiency in pluripotent cells yields somatic cells with DNA methylation abnormalities and pluripotency induction defects

Rachel Lasry,[1,4] Noam Maoz,[1,4] Albert W. Cheng,[2,3] Nataly Yom Tov,[1] Elisabeth Kulenkampff,[2,3] Meir Azagury,[1] Hui Yang,[2,3] Cora Ople,[2,3] Styliani Markoulaki,[2,3] Dina A. Faddah,[2,3] Kirill Makedonski,[1] Dana Orzech,[1] Ofra Sabag,[1] Rudolf Jaenisch,[2,3] and Yosef Buganim[1,*]

[1]Department of Developmental Biology and Cancer Research, The Institute for Medical Research Israel-Canada, The Hebrew University-Hadassah Medical School, Jerusalem 91120, Israel
[2]Whitehead Institute for Biomedical Research, Cambridge, MA 02142, USA
[3]Department of Biology, Massachusetts Institute of Technology, Cambridge, MA 02139, USA
[4]These authors contributed equally
*Correspondence: yossib@ekmd.huji.ac.il
https://doi.org/10.1016/j.stemcr.2023.09.009

SUMMARY

A complete knockout of a single key pluripotency gene may drastically affect embryonic stem cell function and epigenetic reprogramming. In contrast, elimination of only one allele of a single pluripotency gene is mostly considered harmless to the cell. To understand whether complex haploinsufficiency exists in pluripotent cells, we simultaneously eliminated a single allele in different combinations of two pluripotency genes (i.e., *Nanog*$^{+/-}$;*Sall4*$^{+/-}$, *Nanog*$^{+/-}$;*Utf1*$^{+/-}$, *Nanog*$^{+/-}$;*Esrrb*$^{+/-}$ and *Sox2*$^{+/-}$;*Sall4*$^{+/-}$). Although these double heterozygous mutant lines similarly contribute to chimeras, fibroblasts derived from these systems show a significant decrease in their ability to induce pluripotency. Tracing the stochastic expression of *Sall4* and *Nanog* at early phases of reprogramming could not explain the seen delay or blockage. Further exploration identifies abnormal methylation around pluripotent and developmental genes in the double heterozygous mutant fibroblasts, which could be rescued by hypomethylating agent or high OSKM levels. This study emphasizes the importance of maintaining two intact alleles for pluripotency induction.

INTRODUCTION

Embryonic development and cell fate induction require appropriate gene dosage for the activation of the regulatory circuits that control cellular identity.

While a complete knockout (KO) of an important gene may be detrimental to the cell as seen for *Oct4* and *Sox2* (Masui et al., 2007; Nichols et al., 1998), a complete KO of other genes such as *Nanog*, while partially maintains the pluripotent state of the cells, and contributes to chimeras, shows a dramatic reduced reprogramming efficiency to induced pluripotent stem cells (iPSCs) by their fibroblast derivatives, which can only be partially overcome by high levels of exogenous OCT4, SOX2, KLF4, and MYC (OSKM) factors (Carter et al., 2014; Schwarz et al., 2014). In contrast, elimination of only one allele in one gene is considered harmless to the cell.

Given this assumption, many fluorescent reporter cell lines have been generated over the years using the knockin/KO (KI/KO) approach, leaving only one intact allele of the targeted gene. Such reporter lines (e.g., *Sox2* [Arnold et al., 2011], *Nanog* [Wernig et al., 2008], and *Utf1* [Morshedi et al., 2013]) are useful to study pluripotency acquisition following reprogramming and nuclear transfer (Buganim et al., 2012, 2014; Boiani et al., 2002). Although one allele elimination is considered safe, there are rare cases when a reduction in expression of approximately 50% is detrimental to the cell, a phenomenon termed haploinsufficiency. Moreover, even when one allele elimination is not detrimental to the cells, our previous study suggest that reduced expression levels of genes such as *Nanog* may result in suboptimal reprogramming, producing low-quality iPSCs (Buganim et al., 2014).

During the maturation phase of the reprogramming process, epigenetic changes happen stochastically to eventually allow expression of the first pluripotent-related genes (Buganim et al., 2013; David and Polo, 2014). Using single-cell analyses, it has been shown that stochastic low expression of pluripotent genes such as *Utf1*, *Esrrb*, *Sall4* (Buganim et al., 2012), and *Nanog* (Polo et al., 2012) can be observed early on in the process in a small fraction of induced cells which is correlated with the low efficiency of reprogramming. The stochastic behavior of the maturation phase ends with the activation of late pluripotent genes such as *Sox2*, *Dppa4*, *Prdm14*, and *Gdf3* (Buganim et al., 2012; Soufi et al., 2012) which unleashes the final deterministic phase, leading to iPSC stabilization (Buganim et al., 2013).

While efforts to understand the link between exogenous pluripotent reprogramming factors, iPSC quality, and efficiency have been substantial (Benchetrit et al., 2019; Buganim et al., 2014; Carey et al., 2011; Sebban and Buganim, 2015), studies focusing on the effect of reduced levels of endogenous pluripotency genes are lacking and mostly rely on single-gene KO or haploid

 © 2023 The Author(s).
This is an open access article under the CC BY license (http://creativecommons.org/licenses/by/4.0/).

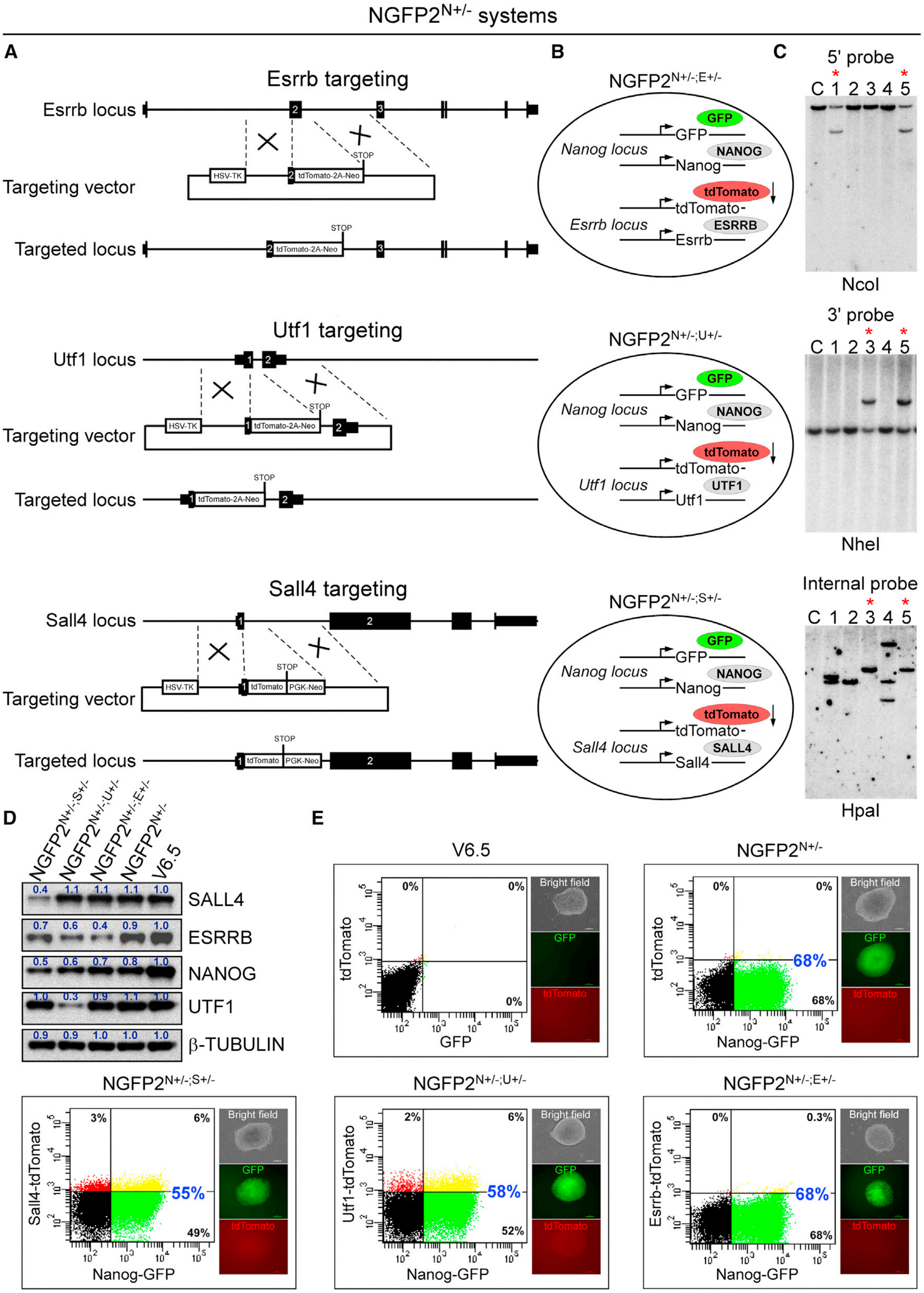

(legend on next page)

embryonic stem cell (ESC) systems (Elling et al., 2019; Leeb and Wutz, 2011). Given this, we sought to examine whether a complex haploinsufficiency (i.e., insufficiency induced by the elimination of one allele in combinations of genes) exists in pluripotent cells and whether and how it may affect their developmental potential and their cells' derivatives.

To address that, we engineered three secondary systems, NGFP2 (Nanog-GFP#2 [Wernig et al., 2008]), NGFP1 (Nanog-GFP#1 [Wernig et al., 2008]), and SGFP1 (Sox2-GFP#1) to incorporate KO of one allele in two different pluripotent genes. These double heterozygous mutant lines include NGFP2 (*Nanog*$^{+/-}$;*Sall4*$^{+/-}$, *Nanog*$^{+/-}$;*Esrrb*$^{+/-}$ and *Nanog*$^{+/-}$;*Utf1*$^{+/-}$), NGFP1 (*Nanog*$^{+/-}$;*Sall4*$^{+/-}$), and SGFP1 (*Sox2*$^{+/-}$;*Sall4*$^{+/-}$). Interestingly, while all double heterozygous mutant lines contributed to chimeras similarly to their parental iPSC controls (i.e., NGFP2 [*Nanog*$^{+/-}$], NGFP1 [*Nanog*$^{+/-}$], and SGFP1 [*Sox2*$^{+/-}$]), multiple derivations of fibroblasts from these lines resulted in poor reprogramming efficiency. This reduced reprogramming efficiency was evident in the nuclear transfer (NT) technique as well.

Tracing the stochastic expression of *Sall4* or *Nanog* along the reprogramming process revealed that only a very small fraction of cells activated these loci, a result that cannot explain the global reprogramming blockage seen in the double heterozygous mutant lines. We then profiled the CpG-rich methylation landscape of fibroblasts derived from SGFP1$^{S2+/-;S4+/-}$ and SGFP1$^{S2+/-}$ control, and noted a clear difference in the methylation levels of multiple developmental and pluripotent loci in the double heterozygous mutant fibroblasts. Accordingly, treating all double heterozygous mutant fibroblasts for 2 days before factor induction with 5-azacytidine rescued the reprogramming blockage and allowed the induction of pluripotency. This study emphasizes the importance of having two intact alleles for proper pluripotency induction and normal embryonic development, and raises a concern regarding the often used KI/KO technique for the purpose of introducing reporters.

RESULTS

Double heterozygous mutant pluripotent cells contribute to chimeras and exhibit modest transcriptional changes

Considering the vital role of functioning core ESC circuitry to pluripotency, we hypothesized that even a slight decrease in the expression of key pluripotency genes could significantly impact the developmental potential of the cells or the ability of their somatic cell derivatives to undergo reprogramming. We focused our research on secondary iPSC systems (i.e., iPSC clones that harbor functional doxycycline (dox)-inducible OSKM factor integrations in their genome), as these systems contribute to chimeras and exhibit stable and reproducible reprogramming efficiency by minimizing cell heterogeneity (Wernig et al., 2008).

We targeted the NGFP2 secondary system, as it already contains a single KI/KO allele of *Nanog* (Wernig et al., 2008). We chose to eliminate a single allele of *Esrrb*, *Utf1*, or *Sall4* as they have all been shown to be important for pluripotency and reprogramming (Buganim et al., 2012; Feng et al., 2009; Tsubooka et al., 2009). To produce a single allele KO and to be able to monitor the activity of the targeted allele, we designed donor vectors that fused, in frame, to the first or second exon a tdTomato reporter (Figures 1A and 1B). To avoid exon skipping and to destabilize the targeted mRNA, polyA was omitted from the targeting vectors. Electroporated colonies were examined for correct targeting by southern blots using external or internal probes (Figure 1C). Overall, we isolated two correctly targeted clones for each combination of manipulated genes: Nanog$^{+/-}$; Esrrb$^{+/-}$ (NGFP2$^{N+/-;E+/-}$, clones# 1 and 5), Nanog$^{+/-}$; Utf1$^{+/-}$ (NGFP2$^{N+/-;U+/-}$, clones# 3 and 5) and Nanog$^{+/-}$; Sall4$^{+/-}$ (NGFP2$^{N+/-;S+/-}$, clones# 3 and 5). To validate the reduced levels of the targeting genes, we cultured the cells in 2i/L medium (GSK3β and MEK inhibitors and Lif) that recapitulates the ground pluripotent state and facilitates gene expression from both alleles (Miyanari and Torres-Padilla, 2012). qPCR and western blot analyses

Figure 1. Generation of double heterozygous mutant NGFP2$^{N+/-}$ iPSC lines

(A and B) Schematic representation of the KI/KO targeting strategy for replacing one allele of *Esrrb*, *Utf1*, or *Sall4* with tdTomato in NGFP2$^{N+/-}$ line. For Esrrb, we targeted exon 2 since it is common to all isoforms of the gene.

(C) Southern blot analyses for NGFP2$^{N+/-}$-targeted iPSC clones demonstrate heterozygous targeting for *Esrrb*, *Utf1*, and *Sall4*. Correctly targeted clones are marked by red asterisks.

(D) Western blot analysis demonstrates a reduction of approximately 50% of the protein levels of the targeted genes (*Esrrb*, *Utf1*, *Nanog*, and *Sall4*) compared with ESC (V6.5) control. Cells were grown in 2i/L condition to facilitate expression from both alleles. Band intensities were quantified using ImageJ, with the quantification values indicated above each corresponding band. Intensities are relative to the V6.5 ESC control band, which was set as a reference value of 1.

(E) Flow cytometry analysis for GFP (*Nanog*) and tdTomato (*Utf1*, *Esrrb*, or *Sall4*) in the various double heterozygous mutant lines that grew under S/L conditions. Representative flow cytometry plots are shown for one experiment out of three independent runs (n = 3). See also Figure S1.

demonstrated a reduction in approximately 50% of the total mRNA or protein levels of all targeted alleles (Figures 1D and S1A), but not in other key pluripotency genes such as *Oct4*, *Sox2*, *Lin28*, *Fbxo15*, and *Fgf4* (Figure S1B). Some further reduction in the protein level of NANOG and ESRRB was seen in NGFP2$^{N+/-;U+/-}$ and NGFP2$^{N+/-;S+/-}$ iPSC lines (Figure 1D) and in the mRNA of the *Dppa3* gene in NGFP2$^{N+/-;S+/-}$ line (Figure S1A). These results suggest that *Nanog* and *Esrrb* are either direct or indirect targets of SALL4 and UTF1 and that *Dppa3* is regulated by SALL4. To test the stability of the targeted alleles, cells grown in either serum/Lif (S/L) or 2i/L conditions were analyzed for GFP and tdTomato activity using flow cytometry. In agreement with the western blot analysis, cells grown under S/L conditions exhibited 68% GFP reporter activity (reporter that was introduced in frame and contains polyA) in NGFP2$^{N+/-}$ control and NGFP2$^{N+/-;E+/-}$ iPSC lines, and 55% and 58% in NGFP2$^{N+/-;S+/-}$ and NGFP2$^{N+/-;U+/-}$ iPSC lines, respectively (Figure 1E). In accordance with our strategy, tdTomato activity for all targeted genes was minor (Figure 1E). Nanog-GFP and tdTomato reporters showed improved activation under 2i/L conditions in all clones, but a reduced percentage remained in the double heterozygous mutant iPSC lines (Figure S1C).

To investigate the impact of eliminating a single allele in two different pluripotent genes on the developmental potential of the cells, we injected the cells into blastocysts and measured their potential to form chimeric mice. A comparable grade of chimerism was noted between all double heterozygous mutant and control iPSC lines, suggesting that elimination of a single allele in these combinations of two genes does not exert a significant developmental barrier (Figure S2A).

Gene expression can distinguish between iPSCs with poor, low, and high quality as assessed by grade of chimerism and 4n complementation assay (Buganim et al., 2014). Thus, we profiled the transcriptome of the three heterozygous mutant lines, as well as the parental NGFP2$^{N+/-}$ cells and wild-type (WT) ESCs (V6.5), grown in either S/L or 2i/L conditions. Pearson correlation heatmap clustered the cells into two main groups based on the culture conditions. Nevertheless, within the S/L group some changes in gene expression were noted in NGFP2$^{N+/-;S+/-}$ and NGFP2$^{N+/-;U+/-}$ compared with NGFP2$^{N+/-;E+/-}$, parental NGFP2$^{N+/-}$, and control WT ESCs (Figure S2B). Given that *Esrrb* has been identified as a downstream target gene of NANOG (Festuccia et al., 2012), it is unsurprising that minimal transcriptional changes were observed between the parental NGFP2$^{N+/-}$ and NGFP2$^{N+/-;E+/-}$ lines. Principal component analysis (PCA) validated the Pearson correlation heatmap, separating S/L conditions from 2i/L conditions by PC1 and NGFP2$^{N+/-;S+/-}$ and NGFP2$^{N+/-;U+/-}$ that were grown under S/L conditions from the rest of the samples by PC2 (Figure S2C). Interestingly, NGFP2$^{N+/-;U+/-}$ grown under S/L conditions, clustered closer to samples that grew under 2i/L conditions as indicated by PC1 (Figure S2C). In contrast, cells grown under 2i/L conditions clustered together with minimal transcriptional changes between them (Figure S2C). Considering the expression differences among the lines grown under S/L conditions, we performed a differential expression analysis ($p < 0.05$, 2-fold change) comparing the control cells with all the double heterozygous mutant iPSC lines. This analysis revealed 1,604 genes with differential expression between the control groups and at least one double heterozygous mutant line (Table S1). Gene Ontology (GO) term analysis for this gene list, using EnrichR (Xie et al., 2021), includes "loss of function of Oct4 in ESCs," "TGFβ regulation," "abnormal heart position," and "abnormal mesendoderm development" (Figure S2D). A gene regulatory network (GRN) constructed using iRegulon identified key pluripotent, mesodermal and neuronal developmental genes, such as *Pou5f1*, *Pqbp1*, *Pax2*, *Bcl11a*, and *Zfp110* (Casademunt et al., 1999; Fotaki et al., 2008; Iwasaki and Thomsen, 2014; Simon et al., 2020), as major regulators of these aberrantly expressed 1,604 genes (Figure S2E). These results suggest that the elimination of one allele of two distinct pluripotent genes, while exhibiting some transcriptional changes under S/L conditions, still maintains a functional pluripotent state with minimal variations in gene expression in the ground pluripotent state.

Fibroblasts derived from NGFP2 double heterozygous mutant iPSC lines fail to induce pluripotency

Given that the reprogramming process involves a stochastic phase of activation of pluripotency genes (Buganim et al., 2012), we hypothesized that mouse embryonic fibroblasts (MEFs) harboring double heterozygous mutant alleles might exhibit reprogramming delay because of difficulties in the activation of the core pluripotency circuitry.

To that end, secondary MEFs were established from all the three NGFP2 double heterozygous mutant lines and control. To initiate reprogramming, MEFs were exposed to dox for 13 days followed by dox withdrawal for 3 more days to stabilize any iPSC colony, and the percentage of Nanog-GFP-positive cells was scored by flow cytometry.

NGFP2$^{N+/-}$ control induced MEFs exhibited the expected approximately 2% of Nanog-GFP-positive cells by the end of the reprogramming, while 2-independent clones from each double heterozygous mutant line showed a complete blockage (Figure 2A). Cell death and proliferation arrest were ruled out, as all double heterozygous mutant and control plates stained equally to crystal violet (Figure 2B), and alkaline phosphatase, albeit to a lesser extent, indicating reprogramming initiation (Figure 2C). By extending dox exposure to 20 days, a small percentage of Nanog-GFP-positive

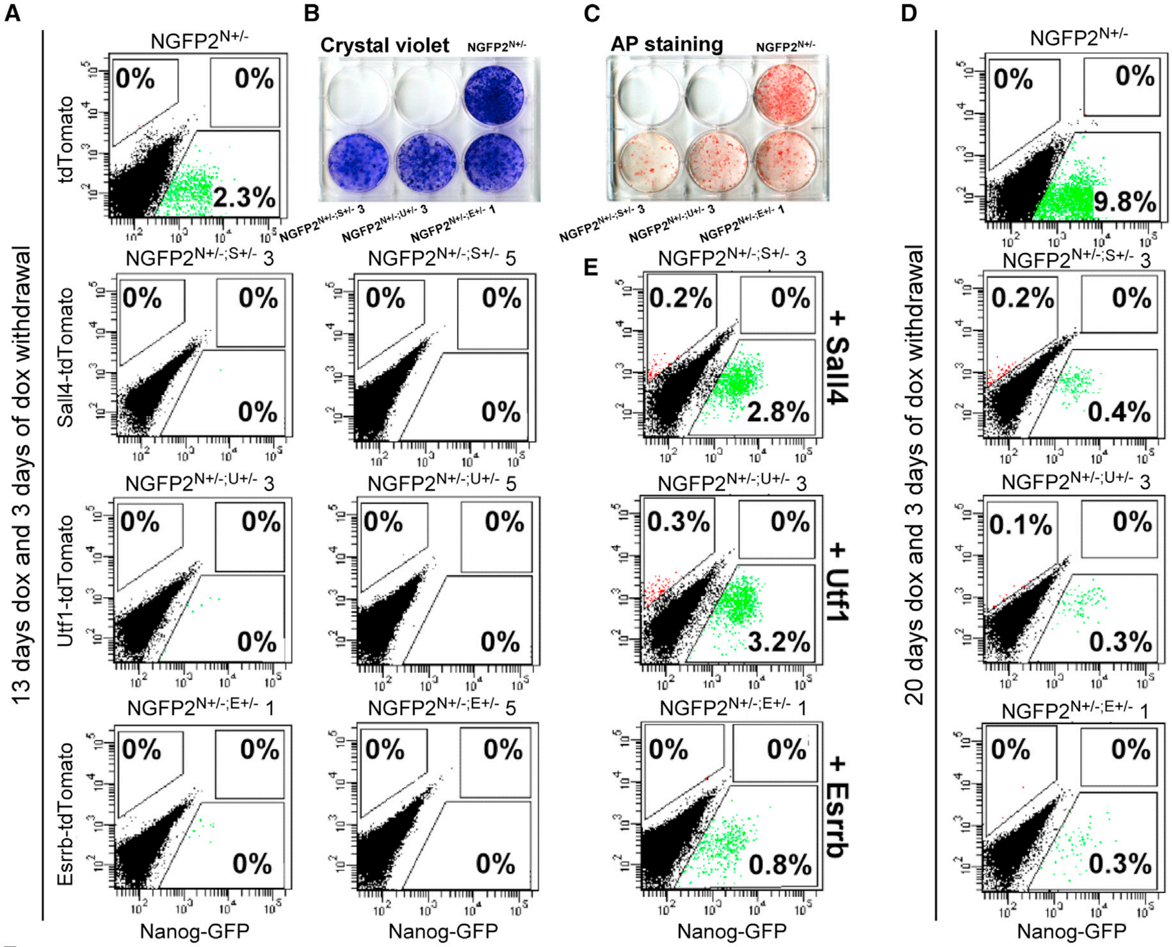

F

Clone #A / Clone #B

MEFs (Clone #)	# of Injected eggs	# & % of formed blastocysts	# & % of established ESC lines
NGFP2N+/-	37	11 (29%)	4 (11%)
NGFP2N+/-;E+/- 1/5	51/45	20 (39%) / 8 (17%)	1 (1.9%) / 1 (2.2%)
NGFP2N+/-;S+/- 3/5	45/80	7 (15%) / 16 (20%)	0 (0%) / 3 (3.8%)
NGFP2N+/-;U+/- 3/5	47/50	6 (12%) / 10 (25%)	0 (0%) / 2 (4%)

G

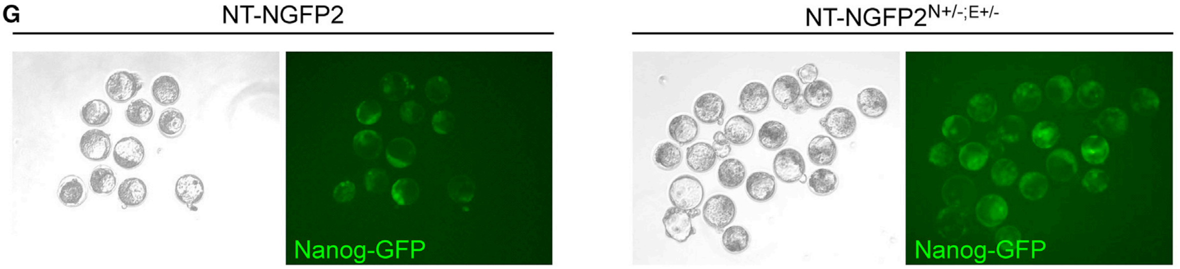

(legend on next page)

cells did emerge in all double heterozygous mutant lines, suggesting that some cells can overcome this blockage when prolonged exposure of OSKM is triggered (Figure 2D).

We then asked whether the reprogramming defect can be rescued by exogenously expressing the targeted genes. Double heterozygous mutant MEFs were transduced with either *Nanog* or with its corresponding targeted gene (i.e., *Sall4*, *Utf1* or *Esrrb*) or with additional viruses encoding for OSK and reprogramming was scored. Both *Nanog* or each of the corresponding factors showed either partial or complete rescue of the reprogramming blockage, while additional OSK further boosted the reprogramming process (Figures 2E, S2F, and S2G). Given that reduced levels of ESRRB was noted in all the double heterozygous mutant iPSC lines (Figure 1D), we asked whether ectopic expression of *Esrrb* can rescue all the mutant MEF lines. While additional expression of *Esrrb* could rescue NGFP2$^{N+/-;E+/-}$ and NGFP2$^{N+/-;U+/-}$, it had only a mild effect, although significant, on NGFP2$^{N+/-;S+/-}$ (Figure S2H). Similarly, ectopic expression of *Sall4* rescued only some of the lines, but not others (Figure S2I). These data suggest that the seen blockage is not specific to a unique allele elimination, but rather it is associated with a broader effect that can be overcome only by high levels of pluripotent factors, such as OSK.

We then explored whether the observed reprogramming blockage is specific to the reprogramming by defined factors or if it would persist in other reprogramming techniques, such as NT. Enucleated eggs were injected with MEF nuclei from each of the three double heterozygous mutant MEF lines and control. Blastocyst formation and establishment of ESC lines were scored. Notably, while all lines exhibited a comparable and expected efficiency in producing blastocysts, the efficiency of ESC line derivation was significantly lower in the double heterozygous mutant lines compared with controls (i.e., 0%–4% vs. 11% in control lines) (Figures 2F and 2G). These results suggest that eliminating two alleles from two distinct key pluripotency genes impacts the somatic nucleus in a manner that hinders its ability to undergo reprogramming to pluripotency.

NGFP2$^{N+/-}$ double heterozygous mutant lines show an early defect in the activation of epithelial markers

We next profiled the transcriptome of the three double heterozygous mutant lines and control lines (i.e., NGFP2$^{N+/-}$ cells, and NGFP2$^{N+/-}$ cells that were infected with empty vector) after 6 days of reprogramming. We chose this time point as it showed a clear reprogramming delay in the double heterozygous mutant plates compared with control plates. NGFP2$^{N+/-}$ MEFs and the parental NGFP2$^{N+/-}$ iPSCs were profiled as well. Hierarchical clustering analysis showed that all the double heterozygous mutant lines clustered together and were different from the control lines (Figure 3A). PCA and scatterplots demonstrate significant transcriptional changes by day 6 of reprogramming between the double heterozygous mutant lines and controls (Figures 3B–3D). Notably, all the double heterozygous mutant lines exhibited minimal transcriptional changes both among themselves and when compared with NGFP2$^{N+/-}$ MEFs, indicating the presence of an early reprogramming defect.

Differential expression analysis between the control groups and all the double heterozygous mutant lines identified 294 genes ($p < 0.05$, 2-fold change) that are upregulated solely in the control groups and 18 genes that are upregulated exclusively in the double heterozygous mutant lines (Figure S3A; Table S1). GO term analysis for the 294 genes of the control groups identified "epithelial cells," "EMT," "tight junction," and "intermediate filament" as the most enriched terms (Figure S3B), suggesting the

Figure 2. NGFP2$^{N+/-}$ double heterozygous mutant MEFs show strong reprogramming inhibition either by OSKM or by NT
(A) Flow cytometry analysis of Nanog-GFP and tdTomato-positive cells for two different clones from each of the NGFP2$^{N+/-}$ double heterozygous mutant induced cells and control after 13 days of dox followed by 3 days of dox withdrawal. Representative flow cytometry plots are shown out of three independent reprogramming runs (n = 3).
(B and C) Crystal violet (B) and alkaline phosphatase (AP) (C) staining of whole reprogramming plates for each of the double heterozygous mutant induced line and control at the end of the reprogramming process. Representative stainings are depicted out of three independent reprogramming runs (n = 3).
(D) Flow cytometry analysis of Nanog-GFP and tdTomato-positive cells for each of the NGFP2$^{N+/-}$ double heterozygous mutant induced cells and control after 20 days of reprogramming. Representative flow cytometry plots are shown out of three independent reprogramming runs (n = 3).
(E) Flow cytometry analysis of Nanog-GFP and tdTomato-positive cells of each of the NGFP2$^{N+/-}$ double heterozygous mutant induced cells and control following overexpression of the targeted gene (*Sall4*, *Utf1*, and *Esrrb*) at the end of the reprogramming process. Representative flow cytometry plots are shown out of three independent reprogramming runs (n = 3).
(F) Table summarizes the efficiency (i.e., blastocyst formation and ESC derivation) of the NT experiments of MEF nuclei of the different double heterozygous mutant NGFP2$^{N+/-}$ lines. NGFP2$^{N+/-}$ (n = 37), NGFP2$^{N+/-;E+/-}$ (n = 96), NGFP2$^{N+/-;s+/-}$ (n = 125), and NGFP2$^{N+/-;u+/-}$ (n = 97). Numbers outside the "/" symbol indicate different targeted clones. For example, "1/5" represents clone #1 and clone #5 for the indicated system.
(G) Representative bright field and green channel images of NGFP2$^{N+/-}$ and NGFP2$^{N+/-;E+/-}$ after NT. See also Figure S2.

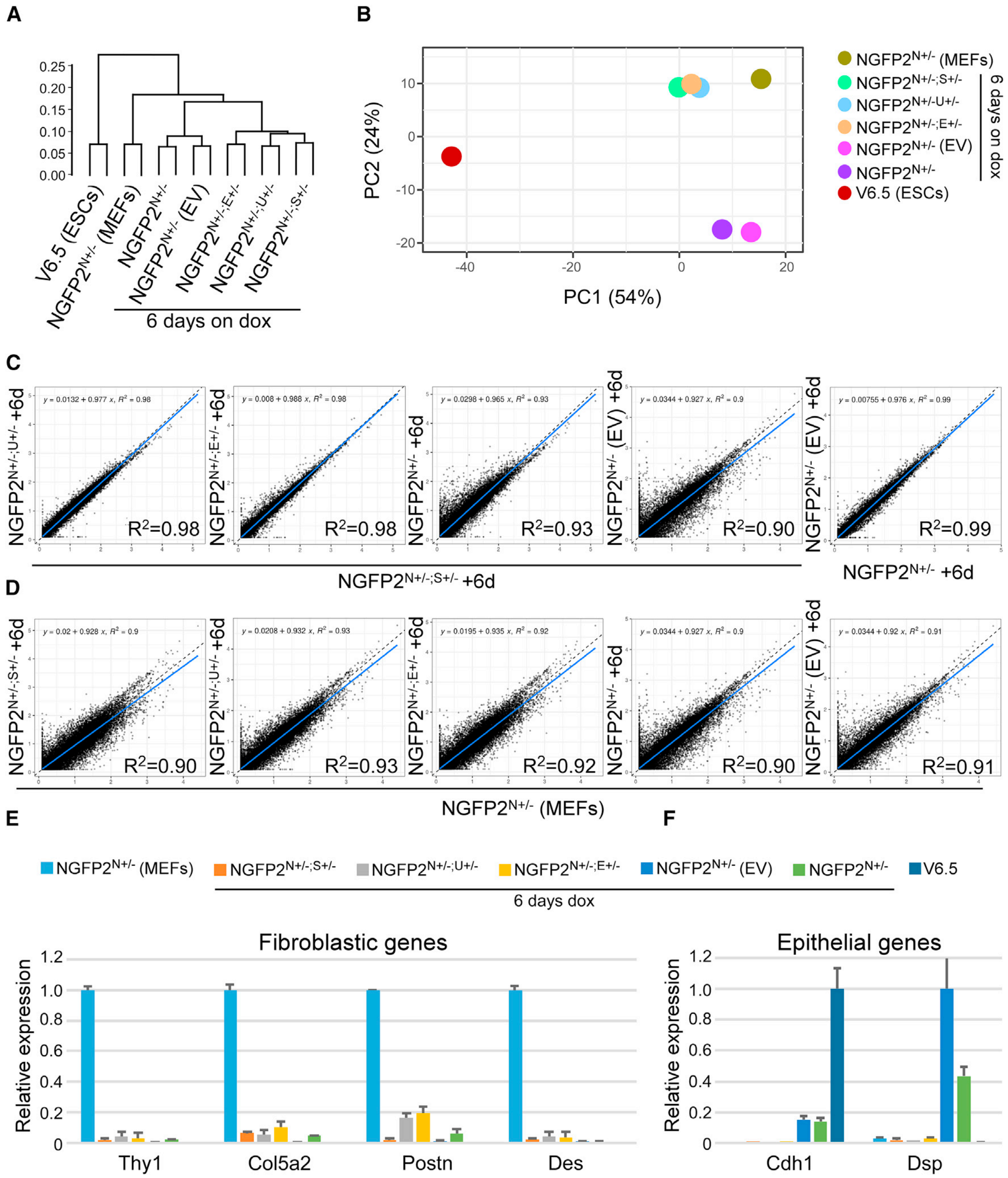

Figure 3. Unbiased comparative transcriptome analyses after 6 days of dox clusters NGFP2$^{N+/-}$ double heterozygote lines far from NGFP2$^{N+/-}$ controls

(A) Hierarchical clustering of global gene expression profiles for two RNA-seq replicates (n = 2) for NGFP2$^{N+/-}$ iPSCs, NGFP2$^{N+/-}$ MEFs and NGFP2$^{N+/-}$, NGFP2$^{N+/-}$ (empty vector [EV]) and the various NGFP2$^{N+/-}$ double heterozygous mutant induced cells (NGFP2$^{N+/-;\ E+/-}$, NGFP2$^{N+/-;\ U+/-}$ and NGFP2$^{N+/-;\ S4+/-}$) after 6 days of reprogramming.

(B) PCA for genes from (A). PC1, 54%; PC2, 24%. Each line is marked by a specific color. The group names correspond with the names in (A). Two replicates from each sample are analyzed (n = 2) and assigned a shared numerical value.

(C and D) Scatterplot graphs compare gene expression between the indicated NGFP2$^{N+/-}$ lines after 6 days of dox and controls. Blue line shows the linear representation of the data. Black line shows the y = x line.

(legend continued on next page)

acquisition of an epithelial identity via mesenchymal to epithelial transition (MET). Accordingly, GRN analysis using iRegulon identified key reprogramming and MET factors such as GLIS1 (Scoville et al., 2017) and GATA2 (Shu et al., 2015) as key regulators for these 294 genes (Figure S3C). GO term analysis of the 18 genes of the double heterozygous mutant lines identified "JUND" as one of the most significant regulators of this gene list and "serotonin receptor signaling" as the most enriched pathway (Figure S3D). Of note, the AP1 family of proteins was previously suggested to act as the safeguard of the fibroblast identity (Jaber et al., 2020; Liu et al., 2015).

Given these analysis, we examined the expression levels of well-known fibroblastic markers (*Thy1*, *Col5a1*, *Postn*, and *Des*) and EMT regulators (*Twist1*, *Zeb1*, *Snai2*, and *Foxc2*), and noticed a comparable downregulation between the control and the double heterozygous mutant lines (Figures 3E and S3E). In contrast, the double heterozygous mutant lines failed to express epithelial genes such as *Cdh1*, *Dsp*, *Epcam*, *Cldn4*, and Cldn7, (Figures 3F and S3F), suggesting late MET blockage.

Reprogramming impairment caused by double heterozygous allele elimination is not restricted to a system or to the identity of the modified alleles

To exclude the possibility that the observed effect is system specific, we used additional secondary iPSC system, NGFP1$^{N+/-}$, which differs in its reprogramming efficiency, dynamics, and factor stoichiometry (Wernig et al., 2008).

As NGFP2$^{N+/-;S+/-}$ demonstrated the strongest delay in pluripotency induction, we thought to eliminate one allele of *Sall4* in NGFP1$^{N+/-}$ as well. Initially, we confirmed by single molecule mRNA-fluorescence *in situ* hybridization (sm-mRNA-FISH) that the strong effect seen in NGFP2$^{N+/-;S+/-}$ is a result of approximately a 50% decrease in the transcript levels of *Sall4* (Figure 4A).

Then, we targeted a tdTomato reporter gene into the *Sall4 locus* of NGFP1$^{N+/-}$ as described above (Figure 4B). Correctly targeted NGFP1$^{N+/-;S+/-}$ iPSC colonies were validated by PCR and western blot (Figures 4C and 4D). We also produced a *Nanog* KO NGFP1$^{N-/-}$ line as a single KO gene control (Figures 4E, 4F, and S4A). Secondary MEFs were produced from NGFP1$^{N+/-}$, NGFP1$^{N+/-;S+/-}$, and NGFP1$^{N-/-}$, which were then exposed to dox for 13 days followed by 3 days of dox removal. Flow cytometry analysis of the various reprogramming plates showed a clear and comparable reduction in the percentage of Nanog-GFP-positive cells in NGFP1$^{N+/-;S+/-}$ and NGFP1$^{N-/-}$-induced cells compared with control NGFP1$^{N+/-}$ cells (Figure 4G). As in the NGFP2$^{N+/-}$ system, exogenous expression of *Nanog* rescued NGFP1$^{N+/-;S+/-}$ double heterozygous mutant cells (Figures 4G and 4H).

We then asked whether the pluripotency induction impairment seen is restricted to combinations that harbor allele elimination of *Nanog*. To that end, we eliminated one allele of *Sall4* in SGFP1$^{S2+/-}$ line, a secondary iPSC system that was generated in our laboratory and contains GFP reporter instead of one allele of *Sox2*. Correctly targeted SGFP1$^{S2+/-;S4+/-}$ iPSC colonies were validated by PCR, western blot, and immunostaining (Figures 4I, 4J, and S4B). As expected, and differently than the NGFP2/1 double heterozygous mutant lines (Figures 1D and S4C), SGFP1$^{S2+/-;S4+/-}$ did not show reduction of ESRRB levels (Figure S4C). Nevertheless, a significant reduction in reprogramming efficiency was noted in SGFP1$^{S2+/-;S4+/-}$ cells compared with SGFP1$^{S2+/-}$ controls (Figures 4K–4M). It is interesting, however, to note that while all the double heterozygous NGFP$^{N+/-}$ lines produced a negligible number of iPSCs following 13 days of reprogramming (i.e., 0.0%–0.2%), the SGFP1$^{S2+/-;S4+/-}$ double heterozygous mutant cells produced approximately 2%–2.5% of iPSCs. This difference can be explained by the levels of the *Oct4* transgene that is much higher in SGFP1$^{S2+/-}$ cells compared with the NGFP$^{N+/-}$ cells (Figure S4D). Taken together, these results suggest that the reprogramming blockage seen in the double heterozygous mutant lines is not specific to a system nor to a combination of eliminated genes' alleles.

Reduced early stochastic expression of the targeted genes cannot explain the reprogramming blockage seen in the double heterozygous mutant lines

Stochastic expression of pluripotency genes during early stages of reprogramming was evident by multiple single-cell studies (Buganim et al., 2012; Guo et al., 2019). Thus, we hypothesized that the lack of two key pluripotency alleles in the double heterozygous mutant cells might impair their ability to pass the early stochastic phase. To explore it, we generated tracing system for *Nanog* and *Sall4*, as they both exhibit high stochastic activity at early stages of reprogramming (Buganim et al., 2012).

We targeted a *2A-EGFP-ERT-CRE-ERT* cassette into the 3′ UTR of *Sall4* or *Nanog* using ESC line that contains a lox-STOP-lox (L-S-L) cassette upstream to a tdTomato reporter gene and M2rtTA transactivator at the *Rosa26* locus

(E and F) qPCR of the indicated fibroblastic genes (E) and epithelial genes (F) in NGFP2$^{N+/-}$ and the different NGFP2$^{N+/-}$ double heterozygous mutant induced cells after 6 days of dox, MEFs, and V6.5 ESCs controls. mRNA levels were normalized to the housekeeping control gene *Gapdh*. Error bars presented as a mean ± SD of two duplicate runs from a typical experiment out of three independent experiments (n = 3). See also Figure S3.

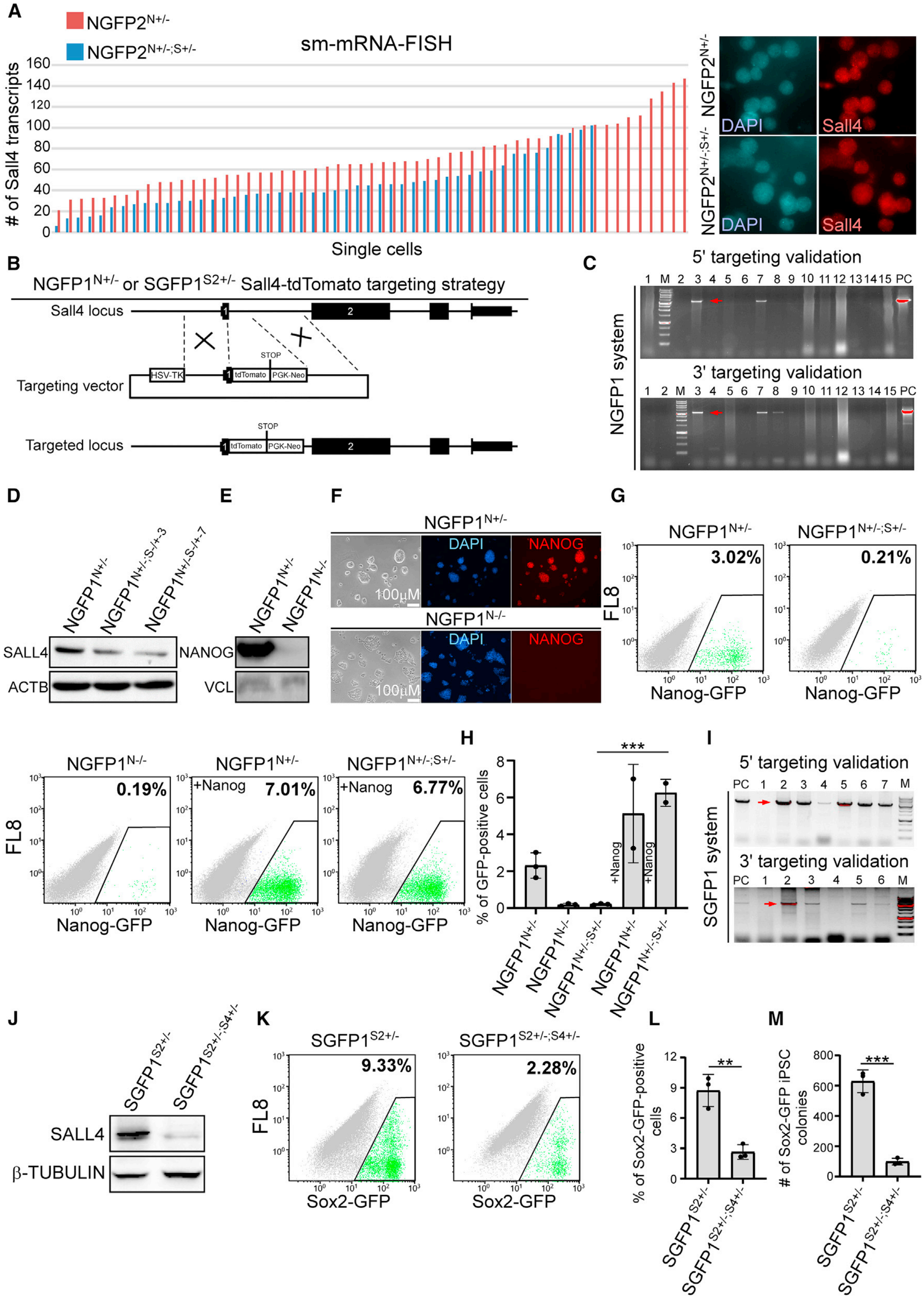

(legend on next page)

(Figures 5A and 5B). Transfected colonies were sorted based on EGFP expression and correct targeting was validated by PCR (Figures 5C and 5D). Correctly targeted ESC clones (i.e., RL8 for *Sall4* and RL9 for *Nanog*) were exposed to tamoxifen (Tam) and the percentage of tdTomato-positive cells was scored by flow cytometry (Figures 5E, 5F, and S5A–S5D), demonstrating high L-S-L cassette removal efficiency.

To correlate the stochastic expression of the targeted alleles to the observed delay, most induced cells should show some activation of the targeted alleles at early time point of reprogramming.

MEFs produced from *Sall4* and *Nanog* tracing ESC systems were transduced with dox-inducible OSKM cassette and tdTomato activation was assessed in the induced cells after 6 days and after 13 days of reprogramming followed by 3 days of dox removal. Only up to 0.24% of the Sall4 tracing cells and up to 0.62% of Nanog tracing cells were tdTomato-positive at day 6 of reprogramming, ruling out the possibility that *Sall4* or *Nanog* stochastic expression early in reprogramming is responsible for the observed blockage (Figures 5G–5I and 5J–5L). In addition, 7.42% of SALL4-2A-EGFP in conjunction with 7.96% of tdTomato-positive cells for the Sall4 tracing system and 2.8% of NANOG-2A-EGFP together with 6.7% of tdTomato-positive cells for the Nanog tracing system at the end of the reprogramming process confirmed successful reprogramming (Figures 5M and 5N). We also explored the ability of NANOG or SALL4-positive cells (i.e., tdTomato cells) to mark reprogrammed cells. On day 6 of reprogramming, tdTomato-positive cells were sorted and reseeded on a feeder layer for continuous reprogramming with dox and Tam. Indeed, both NANOG and SALL4 demonstrated significant enrichment for reprogrammed cells (Figures S5E–S5H). In conclusion, this set of experiments, challenges the notion that reduced stochastic expression of the targeted pluripotent alleles is responsible for the early reprogramming blockage.

Methylation abnormalities in the double heterozygous mutant fibroblasts is correlated with reprogramming impairment

The fact that additional exogenous expression of OSK factors rescued the phenotype of the double heterozygous mutant cells (Figure S2G) suggests that epigenetic abnormalities, rather than the elimination of the targeted alleles themselves, are responsible for the observed blockage. Given the crucial role of DNA methylation in reprogramming, we hypothesized that the double heterozygous mutant MEFs might harbor abnormal DNA methylation that hinders their ability to undergo reprogramming. To test this hypothesis, SGFP1$^{S2+/-;S4+/-}$ MEFs and control SGFP1$^{S2+/-}$ MEFs were subjected to reduced representation bisulfite sequencing (RRBS).

Methylation analysis revealed that the two MEF lines are very similar in regard to their CpG-enriched methylation landscape, suggesting that overall the double heterozygous mutant cells harbor a correct fibroblastic methylation landscape, comprising of approximately 1,900,000 sites, that are shared with the control MEFs. However, read counts did vary between samples and so did reads per site, clustering them as two different groups (Figure 6A). Differentially methylated regions (DMRs) were defined as CpG sites of consecutive tiles that are 100-bp long in size, include at

Figure 4. NGFP1$^{N+/-}$ double heterozygous mutant MEFs and Nanog KO MEFs show strong reprogramming inhibition

(A) sm-mRNA-FISH directed toward *Sall4* transcripts in 57 NGFP2$^{N+/-}$ single iPS cells (n = 57) and 49 NGFP2$^{N+/-;\ S+/-}$ single iPS cells (n = 49).

(B) Schematic representation of the KI/KO targeting strategy for replacing one allele of *Sall4* with tdTomato in NGFP1$^{N+/-}$ and SGFP1$^{S2+/-}$.

(C) PCR analyses for transfected NGFP1$^{N+/-}$ iPSC clones demonstrate correct targeting events (red arrows).

(D) Western blot analysis demonstrates a reduction of approximately 50% of the protein levels of SALL4 compared with NGFP1$^{N+/-}$ controls.

(E and F) NGFP1$^{N+/-}$ iPSCs were transfected with CRISPR/Cas9 and gRNA against *Nanog* to produce *Nanog* KO NGFP1$^{N-/-}$ line. Western blot analysis (E) and immunostaining (F) demonstrate a complete loss of NANOG in the KO line.

(G) Flow cytometry analysis of Nanog-GFP-positive cells in NGFP1$^{N+/-}$, NGFP1$^{N+/-;S+/-}$, NGFP1$^{N-/-}$ and following overexpression of *Nanog* after 13 days of dox followed by 3 days of dox removal.

(H) Graph displays the percentages of Nanog-GFP-positive cells following 13 days of dox and 3 days of dox removal in the indicated lines, or after *Nanog* overexpression. Data are derived from three independent reprogramming experiments (n = 3) or two for *Nanog* overexpression (n = 2). ***p = 0.0006 using a two-tailed unpaired t test calculated by GraphPad Prism (8.3.0).

(I) PCR validation for SGFP1$^{S2+/-;\ S4+/-}$ clones. Red arrows mark targeting events.

(J) Western blot analysis detects SALL4 levels in SGFP1$^{S2+/-}$ and SGFP1$^{S2+/-;\ S4+/-}$ iPSCs.

(K) Flow cytometry analysis of Sox2-GFP-positive cells for SGFP1$^{S2+/-}$ and SGFP1$^{S2+/-;\ S4+/-}$ after 13 days of dox and 3 days of dox withdrawal.

(L and M) Graphs display the percentages (L) or colony number (M) of Sox2-GFP-positive cells for SGFP1$^{S2+/-}$ and SGFP1$^{S2+/-;S4+/-}$ after 13 days of dox and 3 days of dox withdrawal. Error bars indicate standard deviation between three independent experiments/replicates (n = 3). **p = 0.0038, ***p = 0.0003 using a two-tailed unpaired t test calculated by GraphPad Prism (8.3.0). See also Figure S4.

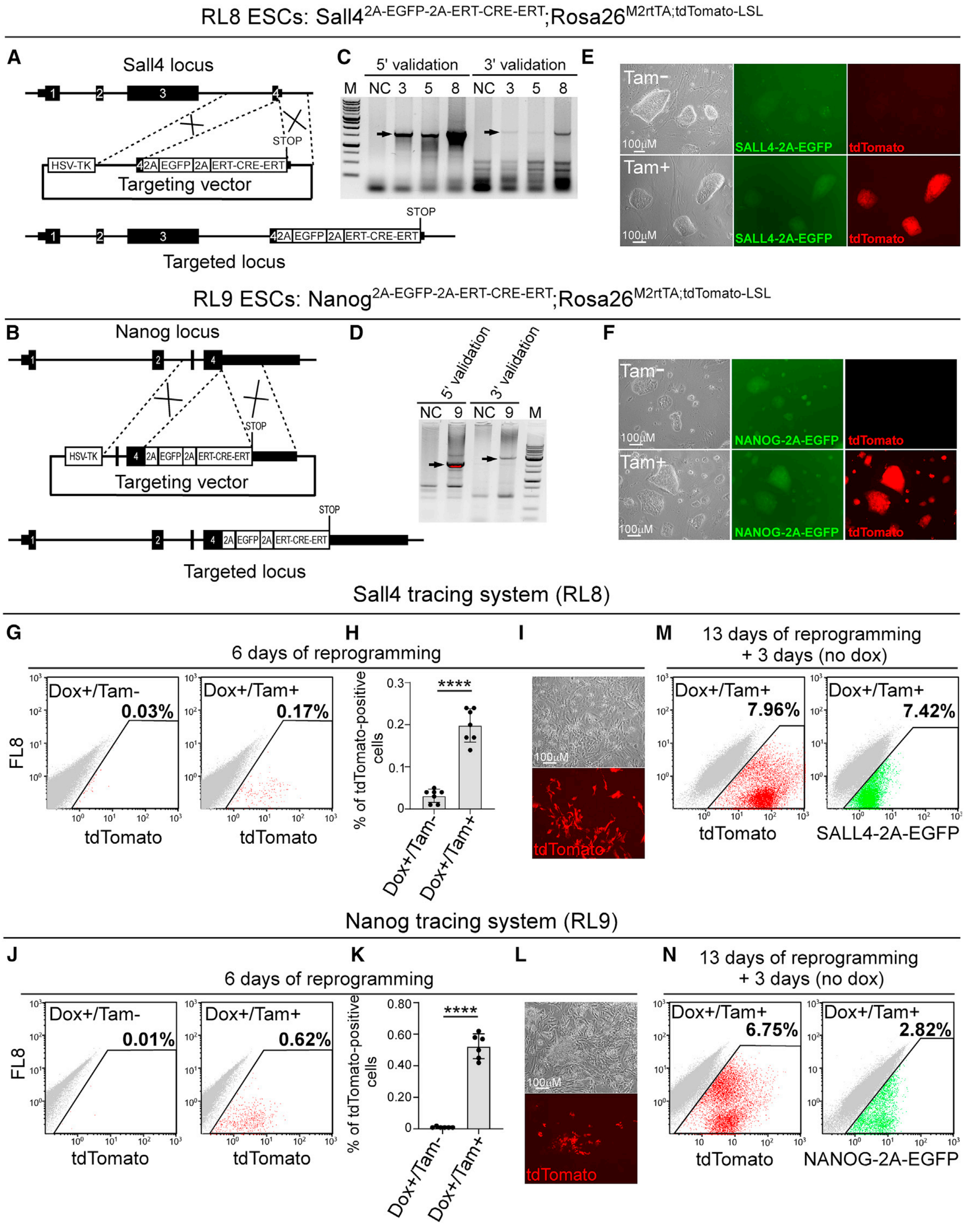

Figure 5. Sall4 and Nanog tracing systems cannot explain the reprogramming blockage observed in the double heterozygous mutant cells

(A and B) Schematic representation of the targeting strategy to introduce a 2A-EGFP-ERT-CRE-ERT cassette into the *Sall4 locus* (A) or into the *Nanog locus* (B).

(C and D) PCR validations for targeted colonies demonstrate correct targeting band size for *Sall4* (C) and for *Nanog* (D) using both 5′ and 3′ regions of the incorporation point. Black arrows depict correct targeting events. NC, negative control.

(legend continued on next page)

least 15 reads and show at least 20% methylation differences between the two MEF lines. All DMRs were adjusted to p value of 1e-3 or lower. This analysis yielded two groups of DMRs: (i) 1,263 tiles that are more methylated and (ii) 1,384 tiles that are less methylated in the double heterozygous mutant MEFs compared with controls (Figures 6B and 6C). We then associated each DMR to its neighboring gene and ran GO term analysis. Interestingly, many of the DMRs were found to be associated with "loss of function of *Oct4*" and are associated with "Hippo signaling" (Figures 6D and 6E), suggesting that the loss of the indicated two pluripotency alleles in the pluripotent state might result in abnormal differentiation and DNA methylation later on in their somatic cell derivatives.

To confirm that DNA methylation abnormalities is responsible for the reprogramming delay, double heterozygous mutant MEFs from all systems were treated for two days with 5-Aza-2′-deoxycytidine (5′azaDC) and reprogramming experiments were carried out. In agreement with the RRBS results, treatment of 5′azaDC rescued the reprogramming defect (Figure 6F).

We then correlated the 1604 differentially expressed genes identified through the comparison between NGFP2$^{N+/-}$ control iPSCs and the double heterozygous mutant iPSC lines (Figure S2D) with the genes affected by methylation in SGFP1$^{S2+/-;S4+/-}$ MEFs. A significant overlap was observed, with 53 genes displaying hypermethylation and 69 genes showing hypomethylation in SGFP1$^{S2+/-;S4+/-}$ MEFs ($p < 0.00001$) (Figures S6A and S6B; Table S1). This overlap was particularly enriched in pathways governing fibroblastic identity, such as "MEFs," "FGF signaling," and "fibrosis," and was further associated with regulation by pluripotency factors such as "OCT4," "TCF3," "SOX2," and "NANOG" (Figures S6C and S6D). GRN analysis conducted for both gene lists identified the pluripotency factor OCT4 as a major regulator of the 53 hypermethylated genes, along with the TGFPβ protein member SMAD1 and the homeobox protein member NKX2-1. Furthermore, the analysis pinpointed on critical early developmental factors such as PAX2, FOXA1, E2F1, and the homeobox protein CDX4 as major regulators for the 69 hypomethylated genes (Figures S6E and S6F). These findings collectively suggest that reduced pluripotency gene levels during the pluripotent state may lead to methylation abnormalities in regions critical for the function of somatic cell derivatives, and this process is mediated by both pluripotent and key developmental regulators.

DISCUSSION

PSCs in 2i/L culture are less affected by differentiation cues due to robust inhibitor-based protection. Conversely, those in S/L conditions are more prone to differentiation signals, resulting in greater transcriptome heterogeneity. In this scenario, any pluripotency gene expression dysregulation can disrupt pluripotency maintenance, potentially affecting somatic cell derivative development.

Here, by using PSCs as a tested model we aimed to understand how reduced levels of pluripotency genes affects cell's function. We deleted a single allele from various combinations of two pluripotency genes (i.e., *Nanog*$^{+/-}$;*Sall4*$^{+/-}$, *Nanog*$^{+/-}$;*Esrrb*$^{+/-}$, *Nanog*$^{+/-}$;*Utf1*$^{+/-}$, and *Sox2*$^{+/-}$;*Sall4*$^{+/-}$) and used different PSC systems to exclude any system-specific effect.

Interestingly, while examination of the developmental potential of the cells did not reveal a significant difference between the double heterozygous mutant cells and their parental controls, fibroblasts derived from the double heterozygous mutant pluripotent cells demonstrated a strong delay in their capability to induce pluripotency either by transcription factors or by NT. The poor reprogramming

(E and F) Representative bright field, RFP, and GFP channel images for the Sall4 (E) or Nanog (F) tracing systems before and after Tam addition.
(G) Flow cytometry analysis of tdTomato-positive RL8 induced cells that were infected with dox-inducible OSKM vectors and exposed to dox with or without Tam for 6 days.
(H) Graph summarizes the percentages of tdTomato-positive cells of the Sall4 tracing system after 6 days of dox with or without Tam. Error bars indicate standard deviation between 7 independent experiments/replicates ($n = 7$). ****$p < 0.0001$ using a two-tailed unpaired t test calculated by GraphPad Prism (8.3.0).
(I) Bright field and RFP channel images of tdTomato-positive cells from the Sall4 tracing system after 6 days of dox and Tam addition.
(J) Flow cytometry analysis of tdTomato-positive RL9 induced cells that were infected with dox-inducible OSKM vectors and exposed to dox with or without Tam for 6 days.
(K) Graph summarizes the percentages of tdTomato-positive cells of the Nanog tracing system after 6 days of dox with or without Tam. Error bars indicate standard deviation between 6 independent experiments/replicates ($n = 6$). ****$p < 0.0001$ using a two-tailed unpaired t test calculated by GraphPad Prism (8.3.0).
(L) Bright field and RFP channel images of tdTomato-positive cells from the Nanog tracing system after 6 days of dox and Tam addition.
(M and N) Flow cytometry analysis of tdTomato and SALL4-2A-EGFP-positive cells (M) or NANOG-2A-EGFP-positive cells (N) after 13 days of OSKM induction in the presence of dox and Tam followed by 3 days of dox withdrawal. Representative flow cytometry plots are shown out of 7 or 6 independent reprogramming runs (n = 7 for Sall4 and n = 6 for Nanog tracing). See also Figure S5.

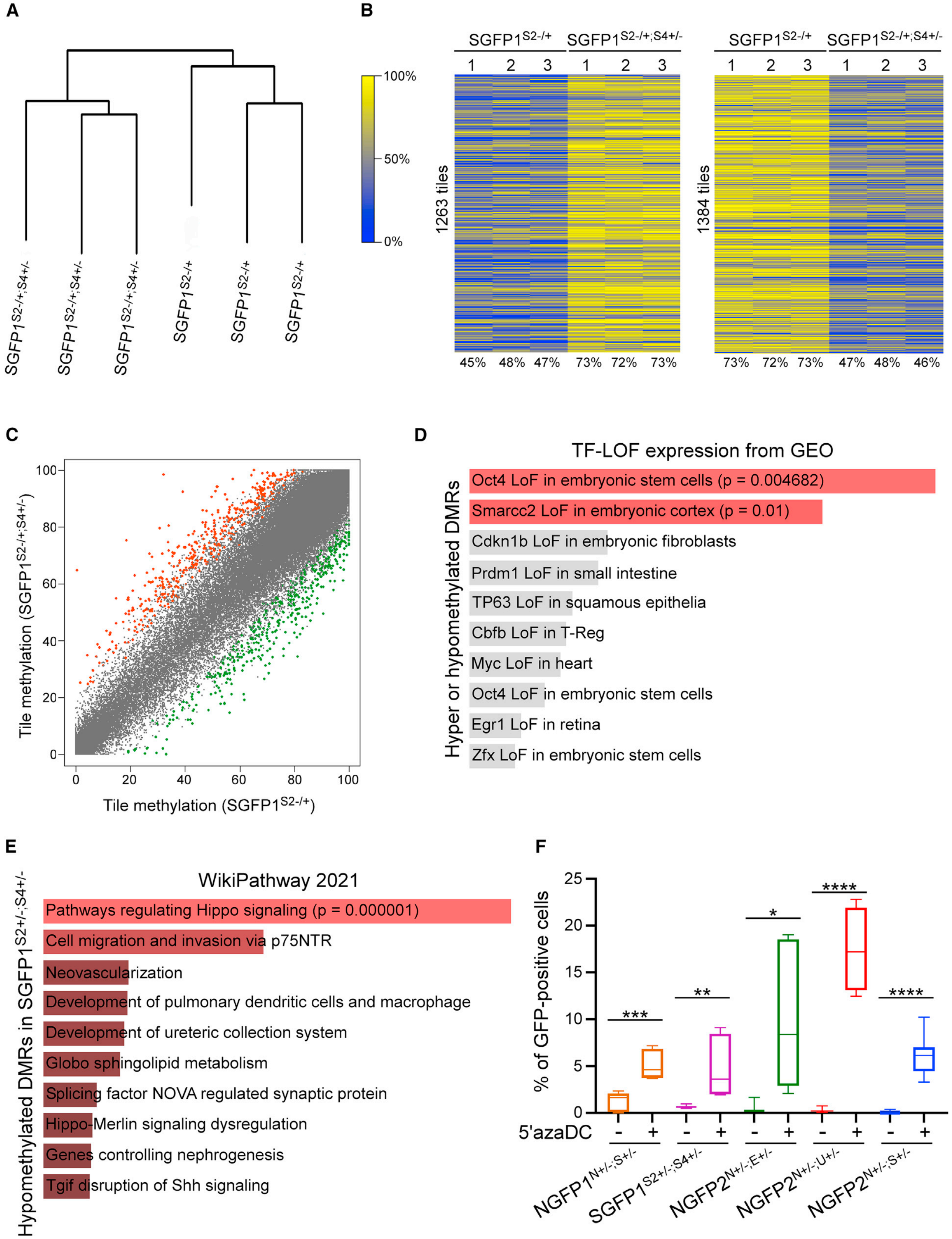

(legend on next page)

efficiency observed between the various pluripotent stem cell systems ranged from a complete blockage at the MET transition (NGFP2 line) to a later blockage at the stabilization step just before the acquisition of pluripotency (NGFP1 and SGFP1 lines).

Given that the affected genes were shown to play a major role during the stochastic phase of the reprogramming process, we examined the possibility that reduced stochastic expression of the targeted genes hinders the capability of the cells to pass the stochastic phase and to induce pluripotency. To support this hypothesis, one should show that the activation of the *Sall4* or *Nanog* allele is a frequent event and occurred in most induced cells at early stages of reprogramming. Using tracing systems for Nanog and Sall4 we show that, only a small number of induced cells could activate the targeted alleles following 6 days of factor induction, suggesting that reduced stochastic expression of these genes is not responsible for the global reprogramming delay seen in the double heterozygous mutant cells.

Additional expression of multiple pluripotent genes (e.g., *Sall4*, *Nanog*, *Utf1*, *Esrrb*, and *OSK*) can either partially or fully rescue the observed blockage; thus, we next hypothesized that epigenetic barrier in the double heterozygous mutant fibroblasts may cause the observed delay. Indeed, CpG-enriched DNA methylation analysis demonstrated a clear difference in the DNA methylation levels in regions within pluripotent and developmental genes between the two fibroblast lines, suggesting that even a 50% reduction in the levels of two pluripotent genes is sufficient to induce aberrant DNA methylation during development. In fact, although *Oct4* expression was unaffected in the iPSCs, GO enrichment analysis of the derived MEFs revealed the loss of Oct4's core pluripotency function. This discrepancy can be attributed to the reduced levels of key pluripotent genes in the iPSCs, including *Nanog*, *Sox2*, *Sall4*, and *Esrrb*, which are known to regulate the core DNA methylation machinery (Adachi et al., 2018; Shanak and Helms, 2020; Tan et al., 2013).

These findings may have implications beyond their impact on pluripotency and reprogramming. Our data indicate that even a 50% reduction in the levels of two pluripotent genes can have significant consequences during embryonic development. This mechanism may provide valuable insights and potential explanations for unresolved cases of spontaneous abortion or improper development.

Fluorescent reporter genes are a widely used tool in science to monitor the activity of a gene, regulatory element, or other elements in the genome. One of the most common approaches to introduce a reporter gene in a locus-specific manner is by the KI/KO approach. In this technique, the genomic sequence of the element of interest is being replaced by the coding sequence of the reporter gene, leaving only one intact allele of the targeted element. Our research highlights the potentially harmful impact of eliminating even a single allele within targeted cells. Consequently, exploring alternative techniques like self-cleavage peptides 2A and the internal ribosome entry site for introducing a reporter gene into the gene of interest, without disrupting the gene's coding sequences, offers notable benefits. Collectively, our findings underscore the importance of maintaining two intact alleles for ensuring optimal cellular functionality.

EXPERIMENTAL PROCEDURES

Resource availability

Corresponding author

Further information and requests for resources and reagents should be directed to and will be fulfilled by the corresponding author, Yosef Buganim (yossib@ekmd.huji.ac.il).

Materials availability

All unique/stable reagents generated in this study are available from the lead contact with a completed Materials Transfer Agreement.

Data and code availability

The accession number for the RNA-seq data for the various NGFP2$^{N+/-}$ double heterozygous mutant and control iPSC lines

Figure 6. DNA methylation abnormalities in the double heterozygous mutant fibroblasts hinder the reprogramming process

(A) Dendrogram for SGFP1$^{S2+/-}$ MEFs and SGFP1$^{S2\ +/-;S4+/-}$ MEFs based on the level of relative change observed at CpG sites with a threshold of 10 reads per site. For each sample, three independent biological replicates are analyzed (n = 3).

(B) Heatmaps display DMRs (20%) in the indicated samples. Each tile (100 bp) is filtered to include at least 15 reads. $p < 0.001$. For each sample, three independent biological replicates are analyzed (n = 3).

(C) Scatterplot analysis (average of 3 replicates, n = 3) shows all the DMRs between SGFP1$^{S2+/-}$ MEFs and SGFP1$^{S2+/-;S4+/-}$ MEFs. Stained tiles are associated with genes that are related to pluripotency and development and are significantly more methylated in SGFP1$^{S2+/-;S4+/-}$ MEFs (red) or in SGFP1$^{S2+/-}$ MEFs (green).

(D and E) GO term enrichment analysis using different categories of EnrichR for hyper- or hypomethylated DMRs (D) or hypomethylated DMRs (E) in SGFP1$^{S2+/-;S4+/-}$ MEFs.

(F) Bar plot graph displays the percentage of GFP-positive cells in the indicated samples after 13 days of dox and 3 days of dox removal with and without prior treatment of 5′azaDC for two days. Boxes indicate 50% (25–75%) and whiskers (5–95%) of all measurements, with middle lines depicting the medians. Data are derived from six to nine independent reprogramming runs (n = 6–9). *p = 0.0127, **p = 0.0069, ***p = 0.0001, ****p < 0.0001 using a two-tailed unpaired t test calculated by GraphPad Prism (8.3.0). See also Figure S6.

is "GEO: GSE182009". The accession number for the RNA-seq for NGFP2$^{N+/-}$ double heterozygous mutant and control MEF lines after 6 days of reprogramming and RRBS for the SGFP1$^{S2+/-}$ and SGFP1$^{S2+/-;S4+/-}$ primary MEFs is "GEO: GSE192655".

Experimental model and subject details

This research was performed in compliance with the joint ethics committee (IACUC) of the Hebrew University and Hadassah Medical Center. The Hebrew University is an AAALAC international accredited institute.

Quantification and statistical analyses

Statistical analysis was performed by 2-tailed unpaired t test calculated by GraphPad Prism (8.3.0). All data are presented mean ± SD. $p < 0.05$ was considered statistically significant. Sufficient sample size was estimated without the use of a power calculation. Data analysis was not blinded.

SUPPLEMENTAL INFORMATION

Supplemental information can be found online at https://doi.org/10.1016/j.stemcr.2023.09.009.

ACKNOWLEDGMENTS

Y.B. is supported by research grants from EMBO Young Investigator Programme (YIP), Howard Hughes Medical Institute International Research Scholar (HHMI, #55008727), Israel Science Foundation (ISF, 161/23), and by a generous gift from Ms. Nadia Guth Biasini. We thank Yuval Nevo and huji core bioinformatics unit for analyzing part of the RNA-seq data.

AUTHOR CONTRIBUTIONS

Y.B. and R.J. conceived the study; Y.B. and R.L. designed the experiments, prepared the figures and wrote the manuscript; Y.B. together with E.K., C.O., and D.F. generated the NGFP2$^{N+/-}$ double heterozygous mutant lines and ran the various reprogramming experiments on NGFP2$^{N+/-}$ lines; R.L. generated the tracing systems for *Nanog* and *Sall4* and the NGFP1$^{N+/-;S+/-}$, NGFP1$^{N-/-}$, and SGFP1$^{S2+/-;S4+/-}$ lines, performed reprogramming experiments on these lines, immunostaining, flow cytometry and 5′azaDC experiments; N.M. prepared the samples for the RNA-seq at day 6 of reprogramming and performed qPCR for the MET genes; N.M. together with N.YT. ran rescue reprogramming experiments; N.M. performed sm-mRNA-FISH; A.W.C. analyzed the RNA-seq data from the various NGFP2$^{N+/-}$ iPSC lines; H.Y. performed NT experiments; S.M. and K.M. injected iPSC lines to produce secondary MEFs and chimeric mice; M.A. helped R.L. to run reprogramming experiments and to analyze the flow cytometry results; D.O. helped running the Esrrb rescue experiments and performed the iRegulon analysis.

DECLARATION OF INTERESTS

The authors declare no competing interests.

DECLARATION OF GENERATIVE AI AND AI-ASSISTED TECHNOLOGIES IN THE WRITING PROCESS.

During the preparation of this work the author(s) used ChatGPT to improve language and readability. After using this tool/service, the authors reviewed and edited the content as needed and take full responsibility for the content of the publication.

Received: April 13, 2023
Revised: September 14, 2023
Accepted: September 15, 2023
Published: October 12, 2023

REFERENCES

Adachi, K., Kopp, W., Wu, G., Heising, S., Greber, B., Stehling, M., Araúzo-Bravo, M.J., Boerno, S.T., Timmermann, B., Vingron, M., and Schöler, H.R. (2018). Esrrb Unlocks Silenced Enhancers for Reprogramming to Naive Pluripotency. Cell Stem Cell *23*, 900–904.

Arnold, K., Sarkar, A., Yram, M.A., Polo, J.M., Bronson, R., Sengupta, S., Seandel, M., Geijsen, N., and Hochedlinger, K. (2011). Sox2(+) adult stem and progenitor cells are important for tissue regeneration and survival of mice. Cell Stem Cell *9*, 317–329.

Benchetrit, H., Jaber, M., Zayat, V., Sebban, S., Pushett, A., Makedonski, K., Zakheim, Z., Radwan, A., Maoz, N., Lasry, R., et al. (2019). Direct Induction of the Three Pre-implantation Blastocyst Cell Types from Fibroblasts. Cell Stem Cell *24*, 983–994.e7.

Boiani, M., Eckardt, S., Schöler, H.R., and McLaughlin, K.J. (2002). Oct4 distribution and level in mouse clones: consequences for pluripotency. Genes Dev *16*, 1209–1219.

Buganim, Y., Faddah, D.A., Cheng, A.W., Itskovich, E., Markoulaki, S., Ganz, K., Klemm, S.L., van Oudenaarden, A., and Jaenisch, R. (2012). Single-cell expression analyses during cellular reprogramming reveal an early stochastic and a late hierarchic phase. Cell *150*, 1209–1222.

Buganim, Y., Faddah, D.A., and Jaenisch, R. (2013). Mechanisms and models of somatic cell reprogramming. Nat. Rev. Genet. *14*, 427–439.

Buganim, Y., Markoulaki, S., van Wietmarschen, N., Hoke, H., Wu, T., Ganz, K., Akhtar-Zaidi, B., He, Y., Abraham, B.J., Porubsky, D., et al. (2014). The developmental potential of iPSCs is greatly influenced by reprogramming factor selection. Cell Stem Cell *15*, 295–309.

Carey, B.W., Markoulaki, S., Hanna, J.H., Faddah, D.A., Buganim, Y., Kim, J., Ganz, K., Steine, E.J., Cassady, J.P., Creyghton, M.P., et al. (2011). Reprogramming factor stoichiometry influences the epigenetic state and biological properties of induced pluripotent stem cells. Cell Stem Cell *9*, 588–598.

Carter, A.C., Davis-Dusenbery, B.N., Koszka, K., Ichida, J.K., and Eggan, K. (2014). Nanog-independent reprogramming to iPSCs with canonical factors. Stem Cell Rep. *2*, 119–126.

Casademunt, E., Carter, B.D., Benzel, I., Frade, J.M., Dechant, G., and Barde, Y.A. (1999). The zinc finger protein NRIF interacts with the neurotrophin receptor p75(NTR) and participates in programmed cell death. EMBO J. *18*, 6050–6061.

David, L., and Polo, J.M. (2014). Phases of reprogramming. Stem Cell Res. *12*, 754–761.

Elling, U., Woods, M., Forment, J.V., Fu, B., Yang, F., Ng, B.L., Vicente, J.R., Adams, D.J., Doe, B., Jackson, S.P., et al. (2019). Derivation and maintenance of mouse haploid embryonic stem cells. Nat. Protoc. *14*, 1991–2014.

Feng, B., Jiang, J., Kraus, P., Ng, J.H., Heng, J.C.D., Chan, Y.S., Yaw, L.P., Zhang, W., Loh, Y.H., Han, J., et al. (2009). Reprogramming of fibroblasts into induced pluripotent stem cells with orphan nuclear receptor Esrrb. Nat. Cell Biol. *11*, 197–203.

Festuccia, N., Osorno, R., Halbritter, F., Karwacki-Neisius, V., Navarro, P., Colby, D., Wong, F., Yates, A., Tomlinson, S.R., and Chambers, I. (2012). Esrrb is a direct Nanog target gene that can substitute for Nanog function in pluripotent cells. Cell Stem Cell *11*, 477–490.

Fotaki, V., Price, D.J., and Mason, J.O. (2008). Newly identified patterns of Pax2 expression in the developing mouse forebrain. BMC Dev. Biol. *8*, 79.

Guo, L., Lin, L., Wang, X., Gao, M., Cao, S., Mai, Y., Wu, F., Kuang, J., Liu, H., Yang, J., et al. (2019). Resolving Cell Fate Decisions during Somatic Cell Reprogramming by Single-Cell RNA-Seq. Mol. Cell *73*, 815–829.e7.

Iwasaki, Y., and Thomsen, G.H. (2014). The splicing factor PQBP1 regulates mesodermal and neural development through FGF signaling. Development *141*, 3740–3751.

Jaber, M., Radwan, A., Loyfer, N., Abdeen, M., Sebban, S., Kolb, T., Zapatka, M., Makedonski, K., Ernst, A., Kaplan, T., et al. (2020). Comparative Parallel Multi-Omics Analysis During the Induction of Pluripotent and Trophectoderm States. Preprint at bioRxiv. https://doi.org/10.1038/s41467-022-31131-8.

Leeb, M., and Wutz, A. (2011). Derivation of haploid embryonic stem cells from mouse embryos. Nature *479*, 131–134.

Liu, J., Han, Q., Peng, T., Peng, M., Wei, B., Li, D., Wang, X., Yu, S., Yang, J., Cao, S., et al. (2015). The oncogene c-Jun impedes somatic cell reprogramming. Nat. Cell Biol. *17*, 856–867.

Masui, S., Nakatake, Y., Toyooka, Y., Shimosato, D., Yagi, R., Takahashi, K., Okochi, H., Okuda, A., Matoba, R., Sharov, A.A., et al. (2007). Pluripotency governed by Sox2 via regulation of Oct3/4 expression in mouse embryonic stem cells. Nat. Cell Biol. *9*, 625–635.

Miyanari, Y., and Torres-Padilla, M.E. (2012). Control of ground-state pluripotency by allelic regulation of Nanog. Nature *483*, 470–473.

Morshedi, A., Soroush Noghabi, M., and Dröge, P. (2013). Use of UTF1 genetic control elements as iPSC reporter. Stem Cell Rev. Rep. *9*, 523–530.

Nichols, J., Zevnik, B., Anastassiadis, K., Niwa, H., Klewe-Nebenius, D., Chambers, I., Schöler, H., and Smith, A. (1998). Formation of pluripotent stem cells in the mammalian embryo depends on the POU transcription factor Oct4. Cell *95*, 379–391.

Polo, J.M., Anderssen, E., Walsh, R.M., Schwarz, B.A., Nefzger, C.M., Lim, S.M., Borkent, M., Apostolou, E., Alaei, S., Cloutier, J., et al. (2012). A molecular roadmap of reprogramming somatic cells into iPS cells. Cell *151*, 1617–1632.

Schwarz, B.A., Bar-Nur, O., Silva, J.C.R., and Hochedlinger, K. (2014). Nanog is dispensable for the generation of induced pluripotent stem cells. Curr. Biol. *24*, 347–350.

Scoville, D.W., Kang, H.S., and Jetten, A.M. (2017). GLIS1-3: emerging roles in reprogramming, stem and progenitor cell differentiation and maintenance. Stem Cell Investig. *4*, 80.

Sebban, S., and Buganim, Y. (2015). Nuclear Reprogramming by Defined Factors: Quantity Versus Quality. Trends Cell Biol. *26*, 65–75.

Shanak, S., and Helms, V. (2020). DNA methylation and the core pluripotency network. Dev. Biol. *464*, 145–160.

Shu, J., Zhang, K., Zhang, M., Yao, A., Shao, S., Du, F., Yang, C., Chen, W., Wu, C., Yang, W., et al. (2015). GATA family members as inducers for cellular reprogramming to pluripotency. Cell Res. *25*, 169–180.

Simon, R., Wiegreffe, C., and Britsch, S. (2020). Bcl11 Transcription Factors Regulate Cortical Development and Function. Front. Mol. Neurosci. *13*, 51.

Soufi, A., Donahue, G., and Zaret, K.S. (2012). Facilitators and impediments of the pluripotency reprogramming factors' initial engagement with the genome. Cell *151*, 994–1004.

Tan, M.H., Au, K.F., Leong, D.E., Foygel, K., Wong, W.H., and Yao, M.W. (2013). An Oct4-Sall4-Nanog network controls developmental progression in the pre-implantation mouse embryo. Mol. Syst. Biol. *9*, 632.

Tsubooka, N., Ichisaka, T., Okita, K., Takahashi, K., Nakagawa, M., and Yamanaka, S. (2009). Roles of Sall4 in the generation of pluripotent stem cells from blastocysts and fibroblasts. Gene Cell. *14*, 683–694.

Wernig, M., Lengner, C.J., Hanna, J., Lodato, M.A., Steine, E., Foreman, R., Staerk, J., Markoulaki, S., and Jaenisch, R. (2008). A drug-inducible transgenic system for direct reprogramming of multiple somatic cell types. Nat. Biotechnol. *26*, 916–924.

Xie, Z., Bailey, A., Kuleshov, M.V., Clarke, D.J.B., Evangelista, J.E., Jenkins, S.L., Lachmann, A., Wojciechowicz, M.L., Kropiwnicki, E., Jagodnik, K.M., et al. (2021). Gene Set Knowledge Discovery with Enrichr. Curr. Protoc. *1*, e90.

Stem Cell Reports, Volume *18*

Supplemental Information

Complex haploinsufficiency in pluripotent cells yields somatic cells with DNA methylation abnormalities and pluripotency induction defects

Rachel Lasry, Noam Maoz, Albert W. Cheng, Nataly Yom Tov, Elisabeth Kulenkampff, Meir Azagury, Hui Yang, Cora Ople, Styliani Markoulaki, Dina A. Faddah, Kirill Makedonski, Dana Orzech, Ofra Sabag, Rudolf Jaenisch, and Yosef Buganim

RESULTS

Figure S1

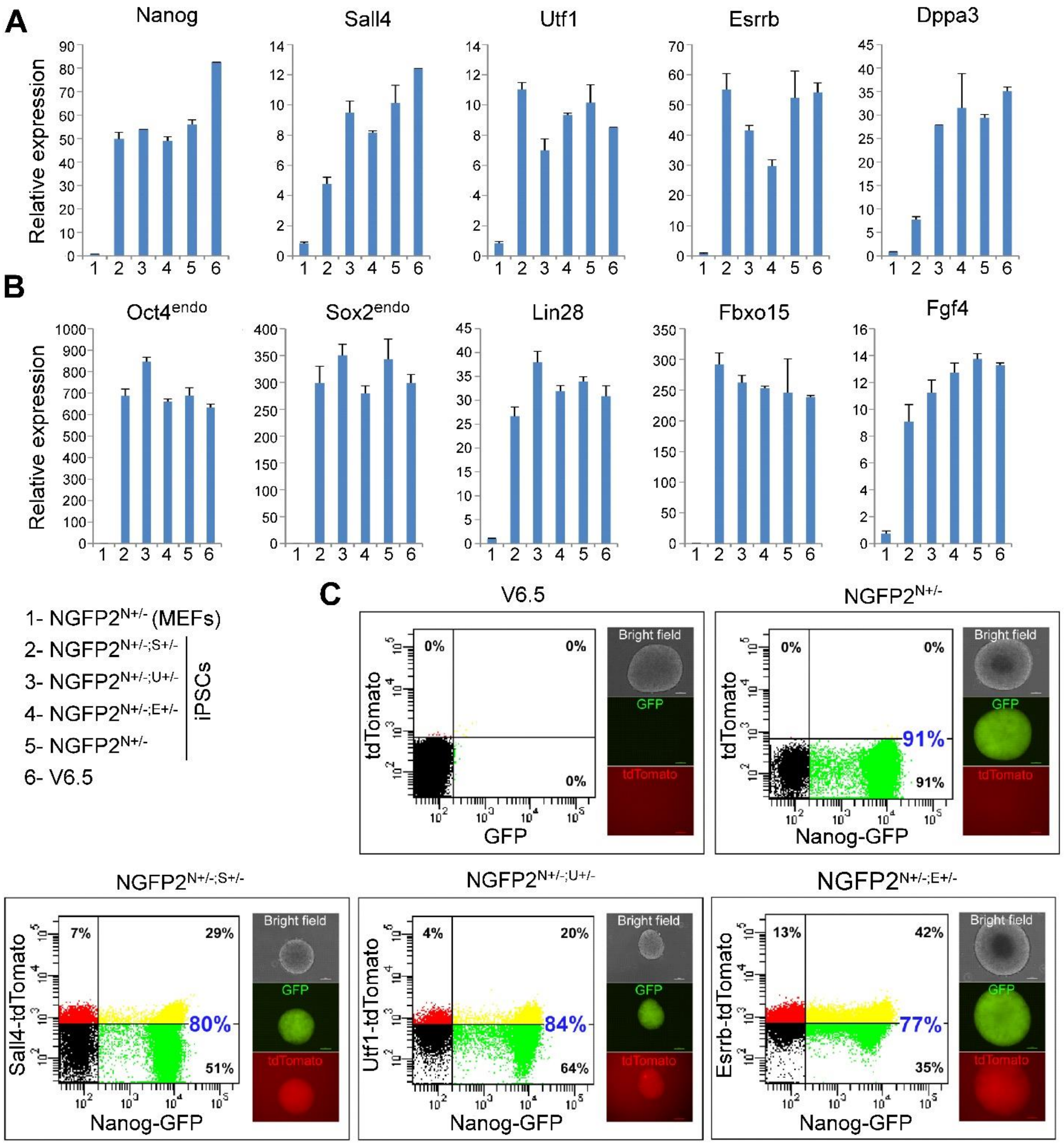

Figure S1. Characterization of the double heterozygous mutant NGFP2$^{N+/-}$ lines, related to main Figure 1. (A, B) qPCR of the indicated genes normalized to the housekeeping control gene *Gapdh* in the various NGFP2$^{N+/-}$ double heterozygous mutant lines, NGFP2$^{N+/-}$ parental line, and ESC (V6.5) and MEF. Error bars presented as a mean ± SD of 2 duplicate runs from a typical experiment out of 3 independent experiments (n= 3). **(C)** Flow cytometry analysis for GFP (*Nanog*) and tdTomato (*Utf1, Esrrb* or *Sall4*) in the various double heterozygous mutant lines that grew under 2i/L conditions. Note that the weak signal of tdTomato is due to the lack of polyA. Representative flow cytometry plots are shown out of three independent runs (n=3).

Figure S2

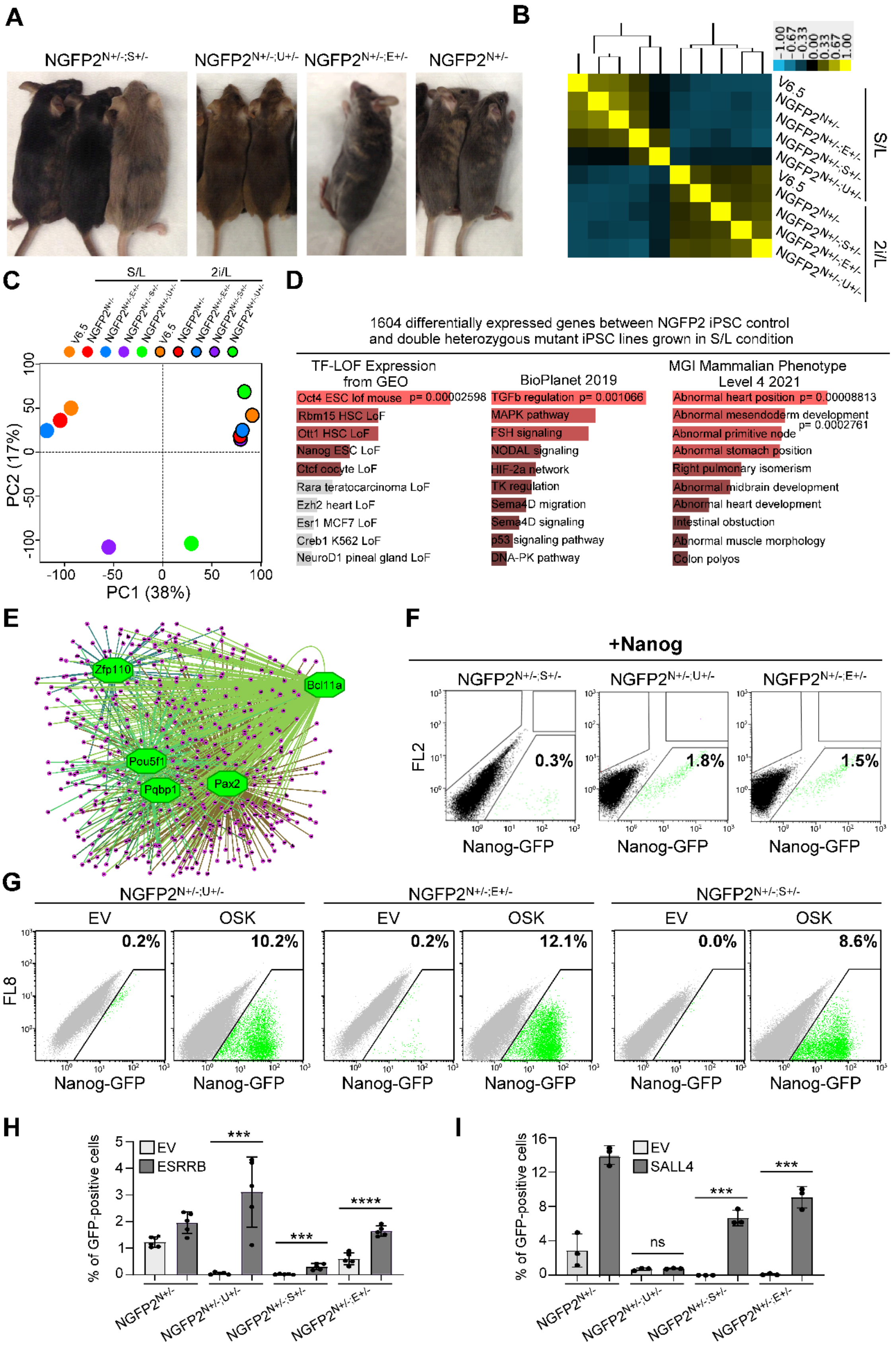
A
NGFP2N+/-;S+/-
NGFP2N+/-;U+/-
NGFP2N+/-;E+/-
NGFP2N+/-
B
C
D
1604 differentially expressed genes between NGFP2 iPSC control
and double heterozygous mutant iPSC lines grown in S/L condition
TF-LOF Expression from GEO
Oct4 ESC lof mouse p= 0.000002598
Rbm15 HSC LoF
Ott1 HSC LoF
Nanog ESC LoF
Ctcf oocyte LoF
Rara teratocarcinoma LoF
Ezh2 heart LoF
Esr1 MCF7 LoF
Creb1 K562 LoF
NeuroD1 pineal gland LoF
BioPlanet 2019
TGFb regulation p= 0.001066
MAPK pathway
FSH signaling
NODAL signaling
HIF-2a network
TK regulation
Sema4D migration
Sema4D signaling
p53 signaling pathway
DNA-PK pathway
MGI Mammalian Phenotype Level 4 2021
Abnormal heart position p= 0.00008813
Abnormal mesendoderm development p= 0.0002761
Abnormal primitive node
Abnormal stomach position
Right pulmonary isomerism
Abnormal midbrain development
Abnormal heart development
Intestinal obstuction
Abnormal muscle morphology
Colon polyos
PC1 (38%)
PC2 (17%)
E
Zfp110
Bcl11a
Pou5f1
Pqbp1
Pax2
F
+Nanog
Nanog-GFP
FL2
G
EV
OSK
FL8
H
% of GFP-positive cells
EV
ESRRB
I
SALL4
ns

Figure S2. The developmental potential and transcriptional profile of NGFP2$^{N+/-}$ double heterozygous mutant lines and rescue reprogramming experiments, related to main Figure 2. (A) Representative images of adult chimeric mice produced by the various NGFP2$^{N+/-}$ double heterozygous mutant iPSC lines and control following blastocyst injection and transplantation into foster mothers. For each line, 30 injected blastocysts were transferred into pseudopregnant females and born mice were analyzed. Representative images show adult chimeric mice for each line and the grade of chimerism. **(B)** Pearson Correlation heatmap and dendrogram of global gene expression profiles for two RNA-seq replicates (n=2) for the indicated NGFP2$^{N+/-}$ iPSC lines and ESC (V6.5) control grown under S/L or 2i/L conditions. Replicate pairs are assigned a shared numerical value. **(C)** Principle component analysis for the indicated samples using 500 most differentially expressed genes among all samples. Two replicates are analyzed for each sample (n=2). PC1, 38%; PC2, 17%. Each line is marked by a specific color. The group names correspond to the names in (B). Cells that were grown in 2i/L are surrounded with black circle. **(D)** Bar graphs show the most enriched GO terms and their p-value, for 1604 genes that demonstrated differential expression between ESC (V6.5)/iPSC (NGFP2$^{N+/-}$) control cells and all double heterozygous mutant iPSC lines, under S/L condition (Table S1), using EnrichR. p-value was calculated using Fisher exact test. **(E)** Gene regulatory network of the 1604 genes from (Table S1) constructed by iRegulon plugin tool in Cytoscape. Transcription factor (FDR < 0.001), Network Enrichment Score (NES) > 3. Green represents key regulators and pink marks regulated genes. Genes with no association were removed from the graph. **(F, G)** Flow cytometry analysis of Nanog-GFP-positive cells for the various NGFP2$^{N+/-}$ double heterozygous mutant induced cells following overexpression of *Nanog* (F) or OSK (G). Reprogramming occurred for 13 days with dox, followed by a 3-day dox removal. OSK indicates *Oct4*, *Sox2* and *Klf4* and EV indicates empty vector. Representative flow cytometry plots are shown out of 3 independent reprogramming runs (n=3). **(H)** Graph shows the percentage of Nanog-GFP-positive cells in the induced cells after 13 days of dox induction and 3 days of dox removal, expressing either empty vector (EV) control or ESRRB. Error bars indicate standard deviation between 5 independent experiments/replicates (n=5). ****p-value< 00001, ***p-value= 0.0009 for Utf1$^{+/-}$, and 0.0006 for Sall4$^{+/-}$ using 2-tailed unpaired t test calculated by GraphPad Prism (8.3.0). **(I)** Graph shows Nanog-GFP-positive cell percentages in the indicated induced cells after 13 days of dox induction and 3 days of dox removal, expressing either empty vector (EV) control or SALL4. Error bars indicate standard deviation between 3 independent experiments/replicates (n=3). ***p-value= 0.0002 for Sall4$^{+/-}$, and 0.0002 for Esrrb$^{+/-}$ using 2-tailed unpaired t test calculated by GraphPad Prism (8.3.0).

Figure S3

Differentially-expressed genes between control cells and all double heterozygous mutant lines at day 6 of reprogramming

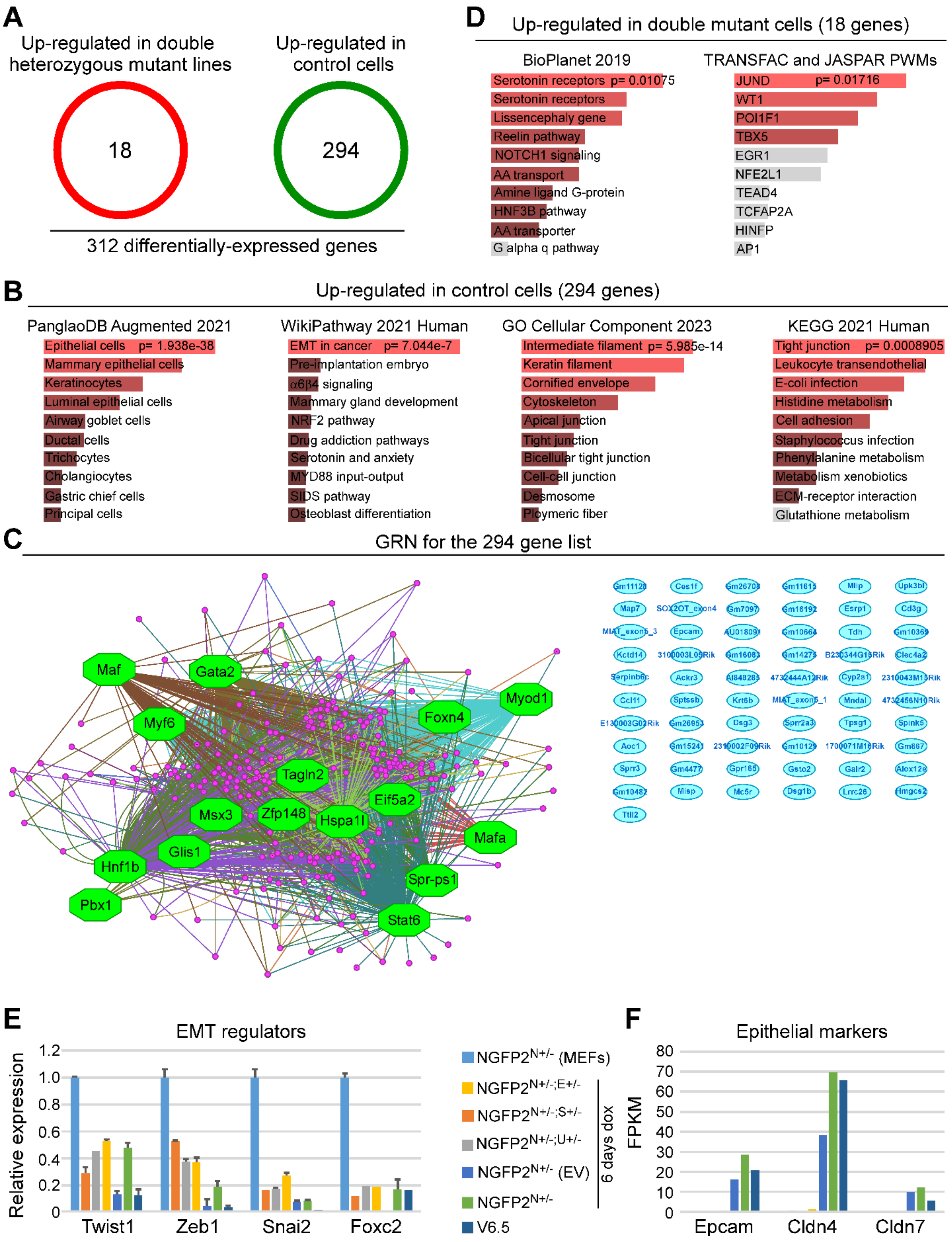

Figure S3. NGFP2$^{N+/-}$ double heterozygous mutant lines fail to activate the epithelial program during reprogramming, related to main Figure 3. (A) Schematic illustration of RNA-seq analysis depicting 18 upregulated genes in NGFP2 double heterozygous mutant lines and 294 upregulated genes in NGFP2$^{N+/-}$ control cells out of 312 differentially expressed genes (p-value< 0.05). **(B)** Bar graphs display the most enriched GO terms and their corresponding p-values for the 294 genes from (A) using EnrichR. The p-values were calculated using Fisher's exact test. **(C)** Gene regulatory network of the 294 genes from (A) constructed by iRegulon plugin tool in Cytoscape. Transcription factor (FDR < 0.001), Network Enrichment Score (NES) > 3. Green represents key regulators, pink marks regulated genes and turquoise depicts genes with no association. **(D)** Bar graphs display the most enriched GO terms and their p-value, for the 18 genes from (A) using EnrichR. p-values were calculated using Fisher exact test. **(E)** qPCR of the indicated EMT genes normalized to housekeeping control gene *Gapdh* in the various NGFP2$^{N+/-}$ double heterozygous mutant induced cells following 6 days of dox and in ESCs (V6.5) and NGFP2$^{N+/-}$ MEF control. Error bars presented as a mean ± SD of 2 duplicate runs (n=2) from a typical experiment out of 3 independent experiments (n=3). **(F)** Graph summarizes the expression level (FPKM- Fragments Per Kilobase Million) of the indicated epithelial genes in the various NGFP2$^{N+/-}$ double heterozygous mutant induced cells after 6 days of dox and in ESCs (V6.5) and NGFP2$^{N+/-}$ MEF control. Expression level of the depicted genes was obtained from the RNA-seq data described in Figure 3.

Figure S4

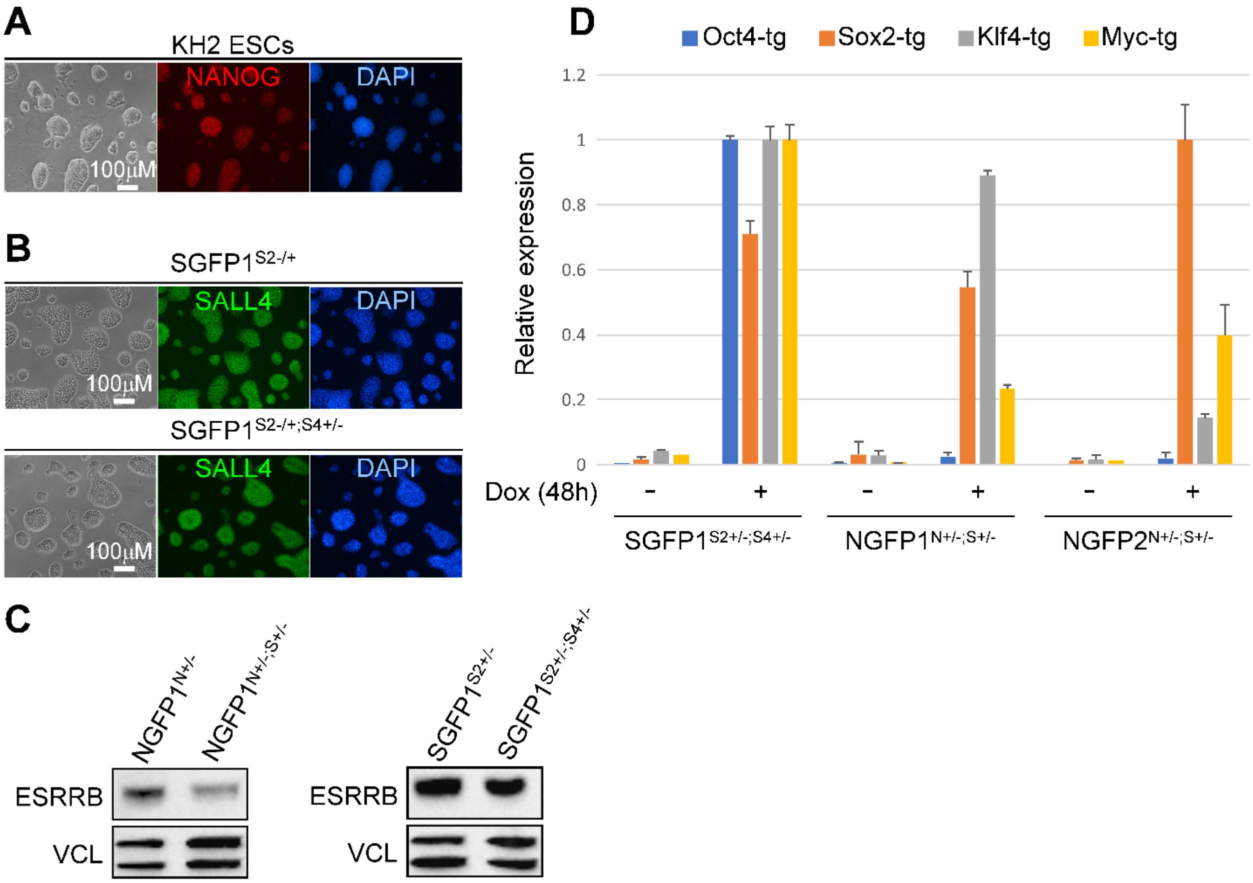
A
KH2 ESCs
NANOG
DAPI
100μM
B
SGFP1S2-/+
SALL4
DAPI
100μM
SGFP1S2-/+;S4+/-
SALL4
DAPI
100μM
C
NGFP1N+/-
NGFP1N+/-;S+/-
SGFP1S2+/-
SGFP1S2+/-;S4+/-
ESRRB
VCL
ESRRB
VCL
D
Oct4-tg
Sox2-tg
Klf4-tg
Myc-tg
Relative expression
1.2
1
0.8
0.6
0.4
0.2
0
Dox (48h)
-
+
-
+
-
+
SGFP1S2+/-;S4+/-
NGFP1N+/-;S+/-
NGFP2N+/-;S+/-

Figure S4. NANOG, SALL4 and ESRRB protein level in targeted iPSC lines and controls, related to main Figure 4. (A) Bright field and immunostaining images for NANOG (red) and DAPI (blue) in KH2 ESCs. **(B)** Bright field and immunostaining images for SALL4 (green) and DAPI (blue) in SGFP1$^{S2+/-}$ and SGFP1$^{S2+/-; S4+/-}$ iPSC lines. **(C)** Western blot analysis of the protein levels of ESRRB in NGFP1$^{N+/-;S+/-}$ and SGFP1$^{S2+/-; S4+/-}$ double heterozygous mutant iPSC lines and in their parental control cells. Cells were grown in 2i/L condition to facilitate expression from both alleles. Vinculin (VCL) was used for loading control. **(D)** qPCR of the indicated OSKM transgenes normalized to housekeeping control gene *Gapdh* in the various double heterozygous mutant MEF lines following 2 days of culture with or without dox. Error bars presented as a mean ± SD of 2 duplicate runs from a typical experiment out of three independent experiments (n=3).

Figure S5

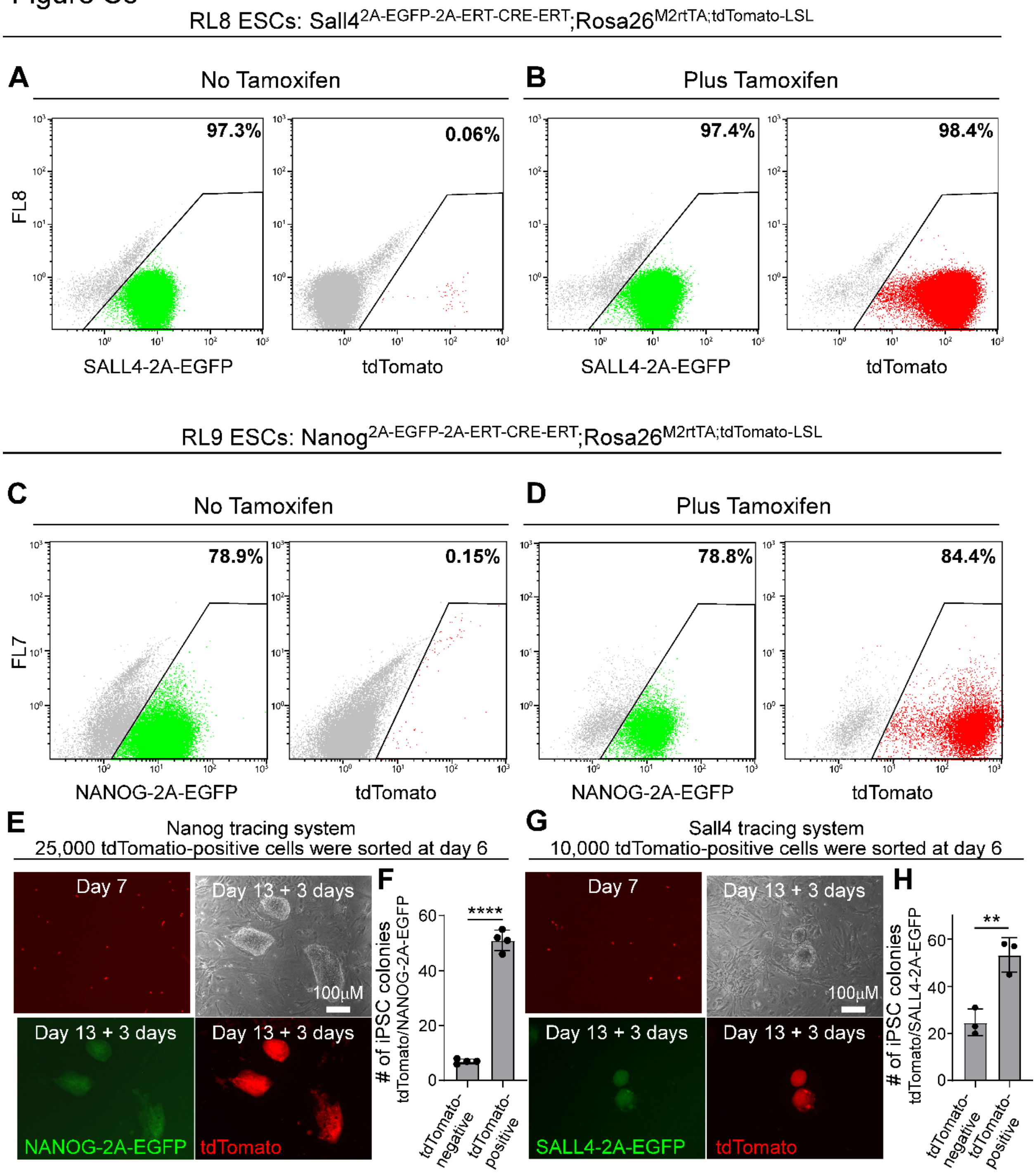
RL8 ESCs: Sall4 2A-EGFP-2A-ERT-CRE-ERT;Rosa26 M2rtTA;tdTomato-LSL
A
No Tamoxifen
B
Plus Tamoxifen
97.3%
0.06%
97.4%
98.4%
FL8
SALL4-2A-EGFP
tdTomato
SALL4-2A-EGFP
tdTomato
RL9 ESCs: Nanog 2A-EGFP-2A-ERT-CRE-ERT;Rosa26 M2rtTA;tdTomato-LSL
C
No Tamoxifen
D
Plus Tamoxifen
78.9%
0.15%
78.8%
84.4%
FL7
NANOG-2A-EGFP
tdTomato
NANOG-2A-EGFP
tdTomato
E
Nanog tracing system
25,000 tdTomatio-positive cells were sorted at day 6
Day 7
Day 13 + 3 days
100μM
Day 13 + 3 days
Day 13 + 3 days
NANOG-2A-EGFP
tdTomato
F
****
# of iPSC colonies
tdTomato/NANOG-2A-EGFP
tdTomato-negative
tdTomato-positive
G
Sall4 tracing system
10,000 tdTomatio-positive cells were sorted at day 6
Day 7
Day 13 + 3 days
100μM
Day 13 + 3 days
Day 13 + 3 days
SALL4-2A-EGFP
tdTomato
H
**
# of iPSC colonies
tdTomato/SALL4-2A-EGFP
tdTomato-negative
tdTomato-positive

Figure S5. Sall4 and Nanog tracing system characterization, related to main Figure 5. (A, B) Flow cytometry analysis for SALL4-2A-EGFP and tdTomato in the targeted ESC clone RL8 before (A) and after tamoxifen addition (B) (48 hours). Representative flow cytometry plots are out of three independent runs (n=3). **(C, D)** Flow cytometry analysis for NANOG-2A-EGFP and tdTomato in the targeted ESC clone RL9 before (C) and after tamoxifen addition (D) (48 hours). Representative flow cytometry plots are shown out of three independent runs (n=3). **(E-H)** MEFs derived from Nanog (E, F) or Sall4 (G, H) tracing system were infected with dox-inducible OSKM lentiviral vectors and reprogrammed in the presence of dox and tamoxifen for 13 days, followed by 3 days of dox removal. On day 6 of reprogramming, tdTomato-positive cells (25,000 cells for Nanog tracing system and 10,000 cells for Sall4 tracing system) were sorted and seeded on feeder-coated wells for continuous reprogramming**. (E, left upper panel)** Representative RFP channel image displays single tdTomato-positive cells from the Nanog tracing system, taken one day after sorting (Day 7). **(E, right and lower panels)** Representative bright field, RFP and green channel images of stable iPSC colonies from tdTomato-positive cells at the end of the reprogramming process. **(F)** Graph summarizes the number of tdTomato/EGFP-positive iPSC colonies generated from tdTomato-negative and tdTomato-positive sorted cells using the *Nanog* tracing system. Error bars indicate standard deviation between 4 independent experiments/replicates (n=4). ****p-value< 0.0001 using 2-tailed unpaired t test calculated by GraphPad Prism (8.3.0). **(G, left upper panel)** Representative RFP channel image shows single tdTomato-positive cells from Sall4 tracing system one day after sorting (Day 7). **(G, right and lower panels)** Representative bright field, RFP and green channel images of stable iPSC colonies from tdTomato-positives cells at the end of the reprogramming process. **(H)** Graph summarizes the number of tdTomato/EGFP-positive iPSC colonies generated from tdTomato-negative and tdTomato-positive sorted cells using the Sall4 tracing system. Error bars indicate standard deviation between 3 independent experiments/replicates (n=3). **p-value= 0.0057 using 2-tailed unpaired t test calculated by GraphPad Prism (8.3.0).

Figure S6

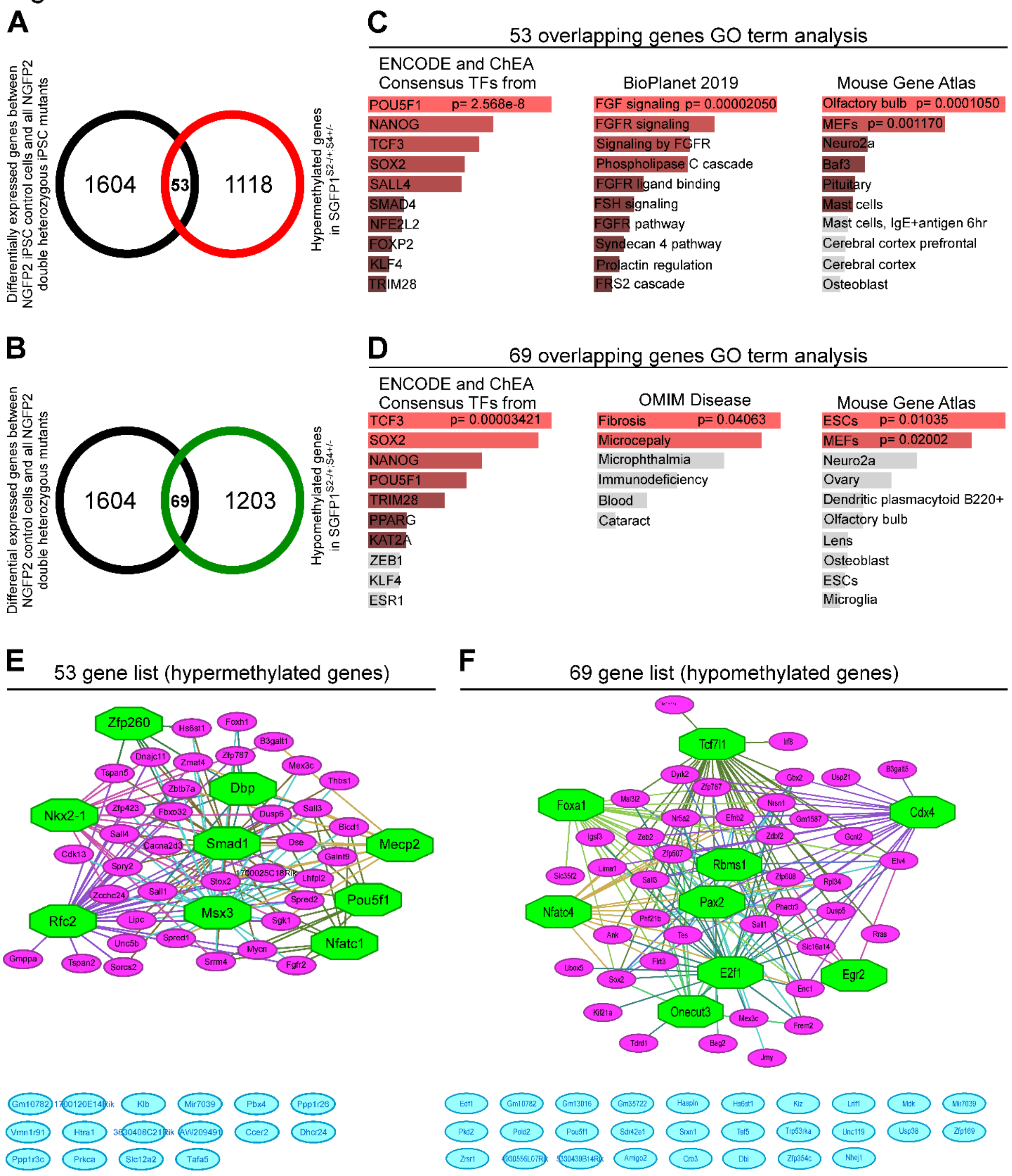
A
Differentially expressed genes between NGFP2 iPSC control cells and all NGFP2 double heterozygous iPSC mutants
1604
53
1118
Hypermethylated genes in SGFP1$^{S2-/+;S4+/-}$
B
Differential expressed genes between NGFP2 control cells and all NGFP2 double heterozygous mutants
1604
69
1203
Hypomethylated genes in SGFP1$^{S2-/+;S4+/-}$
C
53 overlapping genes GO term analysis
ENCODE and ChEA Consensus TFs from
POU5F1 p= 2.568e-8
NANOG
TCF3
SOX2
SALL4
SMAD4
NFE2L2
FOXP2
KLF4
TRIM28
BioPlanet 2019
FGF signaling p= 0.00002050
FGFR signaling
Signaling by FGFR
Phospholipase C cascade
FGFR ligand binding
FSH signaling
FGFR pathway
Syndecan 4 pathway
Prolactin regulation
FRS2 cascade
Mouse Gene Atlas
Olfactory bulb p= 0.0001050
MEFs p= 0.001170
Neuro2a
Baf3
Pituitary
Mast cells
Mast cells, IgE+antigen 6hr
Cerebral cortex prefrontal
Cerebral cortex
Osteoblast
D
69 overlapping genes GO term analysis
ENCODE and ChEA Consensus TFs from
TCF3 p= 0.00003421
SOX2
NANOG
POU5F1
TRIM28
PPARG
KAT2A
ZEB1
KLF4
ESR1
OMIM Disease
Fibrosis p= 0.04063
Microcepaly
Microphthalmia
Immunodeficiency
Blood
Cataract
Mouse Gene Atlas
ESCs p= 0.01035
MEFs p= 0.02002
Neuro2a
Ovary
Dendritic plasmacytoid B220+
Olfactory bulb
Lens
Osteoblast
ESCs
Microglia
E
53 gene list (hypermethylated genes)
Zfp260
Dbp
Nkx2-1
Smad1
Mecp2
Msx3
Pou5f1
Rfc2
Nfatc1
F
69 gene list (hypomethylated genes)
Tcf7l1
Foxa1
Cdx4
Rbms1
Pax2
Nfatc4
E2f1
Egr2
Onecut3

Figure S6. The specific transcriptome of NGFP2$^{N+/-}$ double heterozygous mutant iPSCs exhibit similarities to SGFP1$^{S2+/-}$ double heterozygous MEF methylation profile, related to main Figure 6. (A) Venn diagram displays the 53 overlapping genes (p-value< 0.00001, Fisher exact test) between the 1604 differentially expressed genes in NGFP2$^{N+/-}$ iPSC control versus all NGFP2 double heterozygous mutant iPSC lines and the 1118 hypermethylated genes in SGFP1$^{S2+/-;S4+/-}$ MEFs versus SGFP1$^{S2+/-}$ control MEFs. **(B)** Venn diagram shows the 69 overlapping genes (p-value< 0.00001, Fisher exact test) between the 1604 differentially expressed genes in NGFP2$^{N+/-}$ iPSC control versus all NGFP2 double heterozygous mutant iPSC lines and the 1203 hypormethylated genes in SGFP1$^{S2+/-;S4+/-}$ MEFs versus SGFP1$^{S2+/-}$ control MEFs. **(C, D)** Bar graphs display the most enriched GO terms and their p-value, for the 53 or 69 genes from (A) and (B), respectively using EnrichR. p-values were calculated using Fisher exact test. **(E,F)** Gene regulatory network of the 53 overlapping genes (E) or 69 overlapping genes (F) from (A) and (B), respectively constructed by iRegulon plugin tool in Cytoscape. Transcription factor (FDR < 0.001), Network Enrichment Score (NES) > 3. Green represents key regulators, pink marks regulated genes and turquoise depicts genes with no association.

EXPERIMENTAL PROCEDURES

Cell culture

Mouse embryonic fibroblasts (MEFs) were isolated as previously described (Wernig et al., 2008). MEFs were grown in DMEM supplemented with 10% fetal bovine serum, 1% non-essential amino acids, 2 mM L-Glutamine and antibiotics. ESCs and iPSCs were grown in S/L medium or 2i/L: DMEM supplemented with 10% fetal bovine serum, 1% non-essential amino acids, 2 mM L-Glutamine, 2X106 units mLif, 0.1 mM β-mercaptoethanol (Sigma) and antibiotics with or without 2i- PD0325901 (1 mM) and CHIR99021 (3 mM) (PeproTech). All the cells were maintained in a humidified incubator at 37°C and 6% CO2. All infections were performed on MEFs (passage 0-2) that were seeded at 50-70% confluency two days before the first infection. During the reprogramming to iPSC, the cells were grown in S/L medium with the addition of 2 μg/ml doxycycline.

Secondary MEF production

Briefly, iPSC lines (NGFP2, NGFP1 and SGFP1 lines) were injected into blastocysts and chimeric embryos were isolated at E13.5. For MEF production, embryos were dissected under the binocular removing internal organs and heads. The remaining body was chopped thoroughly by scalpels and exposed to 1ml Tripsin-EDTA (0.25%, GIBCO) for 30 minutes at 37°C. Following that, 10 mL of DMEM medium containing 10%FBS was added to the plate and the chopped tissue was subjected to thorough and intensive pipetting resulting in a relatively homogeneous mix of cells. Each chopped embryo was seeded in 15cm plate and cells were cultured in DMEM supplemented with 10%FBS, 2mM L-glutamine, and antibiotics until the plate was full. Puromycin (2 μg/ml) was added to each 15cm plate for positive selection for NGFP2, NGFP1 and SGFP1 MEFs, eliminating only the host cells.

Immunostaining and Western blot

Cells were fixed in 4% paraformaldehyde (in PBS) for 20 minutes. The cells were rinsed 3 times with PBS and blocked for 1hr with PBS containing 0.1% Triton X-100 and 5% FBS. The cells were incubated overnight with primary antibodies (1:200) in 4C. The antibodies are: anti-SALL4 (Abcam, ab29112, 1:500) and anti-NANOG (Bethyl, A300-379A, 1:500) in PBS containing 0.1% Triton X-100 and 1%FBS. The next day, the cells were washed 3 times and incubated for 1hr with relevant (Alexa, 1:500) secondary antibody in PBS containing 0.1% Triton X-100 and 1% FBS. DAPI (1:1000 dilution) was added 10 minutes before the end of incubation. For western blot, cell pellets were lysed on ice in lysis buffer (20 mM Tris-HCl, pH 8, 1mM EDTA pH 8, 0.5% Nonidet P-40, 150mM NaCl, 10% glycerol, 1mM, protease inhibitors (Roche Diagnostics)

for 10 min, supernatant were collected and 40μg protein were suspended with sample buffer and boiled for or 5 min at 100C, and subjected to western blot analysis. Primary antibodies: anti-SALL4 (Abcam, ab29112, 1:500), anti-NANOG (Bethyl, A300-379A, 1:500), anti-ESRRB (Perseus proteomics, PP-H6705-00, 1:500), anti-UTF1 (Abcam, ab24273, 1:500), anti-ACTB (Santa cruz, sc-1616, 1:500), anti-β-TUBULIN (Abcam, ab179513, 1:500), anti-VCT (Abcam, ab129002, 1:500). Blots were probed with anti-mouse, anti-goat or anti-rabbit IgG-HRP secondary antibody (1:10,000) and visualized using ECL detection kit.

Quantitative real-time PCR

Total RNA was isolated using the Macherey-Nagel kit (Ornat). 500–2000 ng of total RNA was reverse transcribed using iScript cDNA Synthesis kit (Bio-Rad). Quantitative PCR analysis was performed in duplicates using 1/100 of the reverse transcription reaction in a StepOnePlus (Applied Biosystems) with SYBR green Fast qPCR Mix (Applied Biosystems). Specific primers flanking an intron were designed for the different genes (see Primer Table S2). All quantitative real-time PCR experiments were repeated at least three times, and the results were normalized to the expression of *Gapdh* and presented as a mean ± standard deviation of two duplicate runs from a typical experiment.

Southern Blot

Southern blot was performed as previously described (Carey et al., 2011). For primer list see Table S2.

FACS analysis

Cells were washed twice with PBS and trypsinized (0.25%) and filtered through mesh paper. Flow cytometry analysis was performed on a Beckman Coulter and analyzed using Kaluza Software. All FACS experiments were repeated at least three times, and the bar graph results are presented as a mean ± standard deviation of two biological duplicate from a typical experiment. Flow cytometry analysis was performed on a Beckman Coulter and analyzed using Kaluza Software.

RNA sequencing

Total RNA was isolated using Rneasy Kit (QIAGEN) and sent to the “Technion Genome Center”, Israel, for library preparation and sequencing.

Cleaning and filtering of raw reads

Raw reads (fastq files) were inspected for quality issues with FastQC (v0.11.2, http://www.bioinformatics.babraham.ac.uk/projects/fastqc/). According to the FastQC report, reads were then trimmed to a length of 50 bases with fastx_trimmer of the FASTX package (version 0.0.13, http://hannonlab.cshl.edu/fastx_toolkit/), and quality-trimmed at both ends, using in-house perl scripts, with a quality threshold of 32. In short, the scripts use a sliding window of 5 base pairs from the read's end and trim one base at a time until the average quality of the window passes the given threshold. Following quality-trimming, adapter sequences were removed by Trim Galore (version 0.3.7, http://www.bioinformatics.babraham.ac.uk/projects/trim_galore/), using the command "trim_galore -a $adseq –length 15" where $adseq is the appropriate adapter sequence. The remaining reads were further filtered to remove very low-quality reads, using the fastq_quality_filter program of the FASTX package, with a quality threshold of 20 at 90 percent or more of the read's positions.

Expression analysis

The cleaned fastq files were mapped to the mouse transcriptome and genome, Ensembl version GRCm38 from Illumina's iGenomes (http://support.illumina.com/sequencing/sequencing_software/igenome.html), using TopHat (v2.0.11), allowing up to 3 mismatches and a total edit distance of 8 (full command: tophat -G Mus_musculus/Ensembl/GRCm38/Annotation/Genes/genes.gtf -N 3 --read-gap-length 5 --read-edit-dist 8 --segment-length 18 --read-realign-edit-dist 5 --b2-i S,1,0.75 --b2-mp 3,1 --b2-score-min L,-0.5,-0.5 Mus_musculus/Ensembl/GRCm38/Sequence/Bowtie2Index/genome clean.fastq). Quantification and normalization were done with the Cufflinks package (v2.2.1). Quantification was done with cuffquant, using the genome bias correction (-b parameter), multi-mapped reads assignment algorithm (-u parameter) and masking for genes of type IG, TR, pseudo, rRNA, tRNA, miRNA, snRNA and snoRNA (-M parameter). Normalization was done with cuffnorm (using output format of Cuffdiff).

Visualization

The R package cummeRbund (version 2.8.2) was used to calculate and draw the figures (such as scatter plots, MA plots, etc.) from the normalized expression values.

Chimera Formation

Blastocyst injections were performed using (C57/Bl6xDBA) B6D2F2 or CB6F1 host embryos. All injected iPSC lines were derived from crosses of 129Sv/Jae to C57/Bl6 mice and could be identified by agouti coat color. Embryos were obtained 24 hr (1 cell stage) or 40 hr (2 cell stage) posthuman chorionic gonadotropin (hCG) hormone priming. Diploid embryos were cultured in EmbryoMax KSOM (Millipore) or Evolve KSOMaa (Zenith Biotech) until they formed blastocysts (94–98 hr after hCG injection) at which point they were placed in a drop of Evolve w/HEPES KSOMaa (Zenith) medium under mineral oil. A flat tip microinjection pipette with an internal diameter of 16 mm (Origio) was used for iPSC injections. Each blastocyst received 8–12 iPSCs. Shortly after injection, blastocysts were transferred to day 2.5 recipient CD1 females (20 blastocysts per female). Pups, when not born naturally, were recovered at day 19.5 by cesarean section and fostered to lactating Balb/c mothers.

Nuclear transfer

Nuclear transfer was performed as described (Wakayama et al., 1998) with modifications. Briefly, metaphase II-arrested oocytes were collected from superovulated B6D2F1 females (8-10 wks) and cumulus cells were removed using hyaluronidase. The oocytes were enucleated in a droplet of HEPES-CZB medium containing 5μg/ml cytochalasin B (CB) using a blunt Piezo-driven pipette. After enucleation, the spindle-free oocytes were washed extensively and maintained in CZB medium up to 2 h before nucleus injection. The CCs from mice (B6D2F1) were aspirated in and out of the injection pipette to remove the cytoplasmic material and then injected into enucleated oocytes. The reconstructed oocytes were cultured in CZB medium for 1 h and then activated for 5-6 h in activation medium containing 10mM Sr^{2+}, 5ng/ml trichostatin A (TSA) and 5μg/ml CB. Following activation, all of the re constructed embryos were cultured in KSOM medium supplemented with 5ng/ml TSA for another 3-4 hours and maintained in KSOM medium with amino acids at 37C under 5% CO_2 in air.

Reduced-representation bisulfite sequencing (RRBS)

DNA was isolated from MEFs and incubated in lysis buffer (25 mM Tris-HCl (pH 8), 2 mM ethylenediaminetetraacetic acid, 0.2% sodium dodecyl sulfate, 200 mM NaCl) supplemented with 300 μg/mL proteinase K (Roche) followed by phenol:chloroform extraction and ethanol precipitation and RRBS libraries were prepared (Boyle et al., 2012) and run on HiSeq 2500 (Illumina) using 100 bp paired-end sequencing.

DNA methylation was analyzed by using 100 bp paired-end sequencing reads from RRBS that were trimmed and quality filtered by trim galore software using default parameters for RRBS. Read alignment (genome build mm10) and extraction of single-base resolution methylation levels were carried out by BSMAP. Differentially methylated regions (DMR) were explored with R methylKit package version 1.18.0 (Akalin et al., 2012). CpG sites featuring less than 10 reads were considered unreliable and discarded from further analysis. CpG sites were then aggregated into consecutive tiles of size 100 bp and a threshold of at least 15 reads per tile was applied. Differential methylation between the two lines, each consisting of three samples, was determined by logistic regression and adjusted p-values are calculated with SLIM (sliding linear model). A threshold of 1E-3 was set for adjusted p-value and a threshold of 20 methylation points was set between the two lines and further explored. DMRs were annotated with Homer (Hypergeometric Optimization of Motif Enrichment) version 4.11.1 (Heinz et al., 2010) and specifically its function annotatePeaks.pl. This function outputs a set of genes affiliated with DMR based on the nearest promoter distance. Heatmaps were created with R package heatmap.2 version 3.1.1 and dendrogram with R package dendextend version 1.15.2 (Galili, 2015).

Figure legends and tables

Table S1. Differential expressed genes and genomic loci between control and double heterozygous mutant lines, related to main Figures 3, 6, and Supplementary Figures S2, S3, S6.

Table S2. primer list used in this study, related to main Figures 3, 4, 5, and Supplementary Figures S1, S3, S4.

Gene	Application	Primer Sequence (5' --> 3')
Gapdh	qPCR analysis of mRNA expression normalization	F: CCTCAACGACCACTTTGTCAAG<br>R: TCTTCCTCTTGTGCTCTTGCTG
Thy1	qPCR analysis of mRNA expression	F: CCAGAACGTCACAGTGCTCA<br>R: AGGTGTTCTGAGCCAGCAG
Col5a2	qPCR analysis of mRNA expression	F: TAGAGGAAGAAAGGGACAAAAAGG<br>R: GTTACAACAGGCACTAATCCTGGTT
Postn	qPCR analysis of mRNA expression	F: ACAACAATCTGGGGCTTTTT<br>R: AATCTGGTTCCCATGGATGA
Des	qPCR analysis of mRNA expression	F: TGGAGCGTGACAACCTGATA<br>R:AAGGCAGCCAAGTTGTTCTC
Cdh1	qPCR analysis of mRNA expression	F: CTCGACACCCGATTCAAAGT<br>R: GGCGTAGACCAAGAAATGGA

Dsp	qPCR analysis of mRNA expression	F: ACCGTCAACGACCAGAACTC<br>R: TTTGCAGCATTTCTTGGATG
Nanog	qPCR analysis of mRNA expression	F: AAACCAGTGGTTGAAGACTAGCAA<br>R: GGTGCTGAGCCCTTCTGAATC
Oct4 endogenous	qPCR analysis of mRNA expression	F: TCAGTGATGCTGTTGATCAGG<br>R: GCTATCTACTGTGTGTCCCAGTC
Sox2 endogenous	qPCR analysis of mRNA expression	F: CCGTTTTCGTGGTCTTGTTT<br>R: TCAACCTGCATGGACATTTT
Lin28	qPCR analysis of mRNA expression	F: GAAGAACATGCAGAAGCGAAGA<br>R: CCGCAGTTGTAGCACCTGTCT
Fbxo15	qPCR analysis of mRNA expression	F: CGAGAATGGTGGACTAGCTTTTG<br>R: GGCCATGGGAATGAATATTTG
Fgf4	qPCR analysis of mRNA expression	F: GCAGACACGAGGGACAGTCT<br>R: ACTCCGAAGATGCTCACCAC
Sall4	qPCR analysis of mRNA expression	F: GCAAGTCACCAGGGCTCTT<br>R: CCTCCTTAGCTGACAGCAATC
Utf1	qPCR analysis of mRNA expression	F: GTCCCTCTCCGCGTTAGC<br>R: GGCAGGTTCGTCATTTTCC
Esrrb	qPCR analysis of mRNA expression	F: CACCTGCTAAAAAGCCATTGACT<br>R: CAACCCCTAGTAGATTCGAGACGAT
Dppa3	qPCR analysis of mRNA expression	F: TCGGATTGAGCAGAGACAAAAA<br>R: TCCCGTTCAAACTCATTTCCTT
Twist1	qPCR analysis of mRNA expression	F: ACGCTGCCCTCGGACAA<br>R: CCTGGCCGCCAGTTTG
Zeb1	qPCR analysis of mRNA expression	F: CCAGGTGTAAGCGCAGAAAG<br>R: TCATCGGAATCTGAATTTGCT
Snai2	qPCR analysis of mRNA expression	F: ATCCTCACCTCGGGAGCATA<br>R: TGCCGACGATGTCCATACAG
Foxc2	qPCR analysis of mRNA expression	F: AGAACAGCATCCGCCACAAC<br>R: GCACTTTCACGAAGCACTCATT
Oct4-transgene	qPCR analysis of transgenic mRNA expression	F : CGCCTGGAGACGCCATCCACGCT<br>R: GTTGGTTCCACCTTCTCCAA
Sox2-transgene	qPCR analysis of transgenic mRNA expression	F: GCCCAGTAGACTGCACATGG<br>R: AGAATACCAGTCAATCTTTCA
Klf4-transgene	qPCR analysis of transgenic mRNA expression	F: CGCCTGGAGACGCCATCCACGCT<br>R: ACGCAGTGTCTTCTCCCTTC
Myc-transgene	qPCR analysis of transgenic mRNA expression	F : TGTCCATTCAAGCAGACGAG<br>R: AGAATACCAGTCAATCTTTCA
Nanog gRNA	gRNA for generating Nanog KO iPSCs	F: CACCGAGAACTATTCTTGCTTACA<br>R: AAACTGTAAGCAAGAATAGTTCTC
Nanog KO	KO validation PCR	F: CGGCTCACTTCCTTCTGACT<br>R: TATTGCTCCGTCCTGTGTCC
Nanog tracing 5 arm	PCR for generating arm for targeting vector	F : TAACAGCTGAAGTACCTCAGCCTCCAGCA<br>R:TAACAGCTGTATTTCACCTGGTGGAGTCACA
Nanog tracing 3 arm	PCR for generating arm for targeting vector	F: GGTACCCCAGCCCCTGGTTTATTTTT<br>R: CCGCGGACCCACACAGCCTCTCAAGT

Nanog gRNA	gRNA tracing	F: CACCGGATTTGAACTCCTGACCTT<br>R: AAACAAGGTCAGGAGTTCAAATCC
Nanog validation 5 arm tracing	PCR analysis of integration into genomic DNA	F: CCACCCCGTGAACTGACT<br>R: CGTCACCGCATGTTAGAAGA
Nanog validation 3 arm tracing	PCR analysis of integration into genomic DNA	F : GGTACCCCAGCCCCTGGTTTATTTTT<br>R : CCCTGTGAGTGGTCAGGAGT
Sall4 tracing 5 arm	PCR for generating arm for targeting vector	F: GTTAACGCAAGGGAGAGCCAGTATT<br>R: GTTAACGCTGACAGCAATCTTATT
Sall4 tracing 3 arm	PCR for generating arm for targeting vector	F: GGTACCCTGATATGCAAGTGATGT<br>R: CCGCGGATACACACAAGCCCGCCTC
Sall4 gRNA	gRNA tracing	F: CACCGGAGGAGAGGAGTCTTCTGC<br>R: AAACGCAGAAGACTCCTCTCCTCC
Sall4 validation 5 arm tracing	PCR analysis of integration into genomic DNA	F: TAATCCAGCCTTGCTCGTCT<br>R: CGTCACCGCATGTTAGAAGA
Sall4 validation 3 arm tracing	PCR analysis of integration into genomic DNA	F: ACAGCTGTCGAGGTACCCTGA<br>R: GTGTGTGTGTGTCCGTCCTC
Nanog-cDNA	Primers used for cloning of cDNA for lentiviral gene overexpression	F: CGCCATCACACTGACATGA<br>R: TGGAAGAAGGAAGGAACCTG
Sall4-cDNA	Primers used for cloning of cDNA for lentiviral gene overexpression	F: GCAAGTCACCAGGGCTCTT<br>R: CCTCCTTAGCTGACAGCAAT
Esrrb-cDNA	Primers used for cloning of cDNA for lentiviral gene overexpression	F: GCTGGAACACCTGAGGGTAA<br>R: GGTCTCCACTTGGATCGTGT
Utf1-cDNA	Primers used for cloning of cDNA for lentiviral gene overexpression	F: CTACCTGGCTCAGGGATGCT<br>R: GACTGGGAGTCGTTTCTGGA
Sall4 gRNA	gRNA for generating Sall4 KI/KO in NGFP1 and SGFP1	F: CACCGCCAGCTCTCCGCGGATGGT<br>R: AAACACCATCCGCGGAGAGCTGGC
Sall4 5arm validation PCR	PCR analysis of integration into genomic DNA	F: CATACACAAAGCCCCAGGTT<br>R: GCGCATGAACTCTTTGATGA
Sall4 3arm validation PCR	PCR analysis of integration into genomic DNA	F: CGGGATCCGAAGTTCCTATT<br>R: AGCTTGCAAAGGGAAAGACA
Utf1 KI/KO targeting 5arm	PCR for generating arm for targeting vector	F: GAACAGGCTTTTGGCTTCAG<br>R: GGCGCTGGGGACGTCCAGGG<br>Product size: 2920 bps
Utf1 KI/KO targeting 3arm	PCR for generating arm for targeting vector	F: GGCCATACCTTCGAATCCTC<br>R: CCAACACCCAAGAGAAGAGG<br>Product size: 1905 bps
Esrrb KI/KO targeting 5arm	PCR for generating arm for targeting vector	F: AGACACAAGGCTGGAGAGGA<br>R: GGTACCGTGGTAGCCAGAGGCAATG<br>Product size : 3050 bps
Esrrb KI/KO targeting 3arm	PCR for generating arm for targeting vector	F: GGGACCTCAAGGTGAAATGA<br>R: TAAGCCCAACACCTGGAAAC<br>Product size: 3400 bps

Sall4 KI/KO targeting 5arm	PCR for generating arm for targeting vector	F: CAGCCTGGGCTACTTGAGAC<br>R: CTCCTCCCAGTTGATGTGCT<br>Product size: 3200 bps
Sall4 KI/KO targeting 3arm	PCR for generating arm for targeting vector	F: TGGTCCACCTGGAACAAAAG<br>R: AGAAGGGAGCTATGGCACAA<br>Product size: 3155 bps

REFERENCES

Akalin, A., Kormaksson, M., Li, S., Garrett-Bakelman, F.E., Figueroa, M.E., Melnick, A., and Mason, C.E. (2012). methylKit: a comprehensive R package for the analysis of genome-wide DNA methylation profiles. Genome Biol *13*, R87.

Boyle, P., Clement, K., Gu, H., Smith, Z.D., Ziller, M., Fostel, J.L., Holmes, L., Meldrim, J., Kelley, F., Gnirke, A., *et al.* (2012). Gel-free multiplexed reduced representation bisulfite sequencing for large-scale DNA methylation profiling. Genome Biol *13*, R92.

Carey, B.W., Markoulaki, S., Hanna, J.H., Faddah, D.A., Buganim, Y., Kim, J., Ganz, K., Steine, E.J., Cassady, J.P., Creyghton, M.P., *et al.* (2011). Reprogramming factor stoichiometry influences the epigenetic state and biological properties of induced pluripotent stem cells. Cell stem cell *9*, 588-598.

Galili, T. (2015). dendextend: an R package for visualizing, adjusting and comparing trees of hierarchical clustering. Bioinformatics *31*, 3718-3720.

Heinz, S., Benner, C., Spann, N., Bertolino, E., Lin, Y.C., Laslo, P., Cheng, J.X., Murre, C., Singh, H., and Glass, C.K. (2010). Simple combinations of lineage-determining transcription factors prime cis-regulatory elements required for macrophage and B cell identities. Mol Cell *38*, 576-589.

Wakayama, T., Perry, A.C., Zuccotti, M., Johnson, K.R., and Yanagimachi, R. (1998). Full-term development of mice from enucleated oocytes injected with cumulus cell nuclei. Nature *394*, 369-374.

Wernig, M., Lengner, C.J., Hanna, J., Lodato, M.A., Steine, E., Foreman, R., Staerk, J., Markoulaki, S., and Jaenisch, R. (2008). A drug-inducible transgenic system for direct reprogramming of multiple somatic cell types. Nat Biotechnol *26*, 916-924.
